# Logic-gated antibody pairs that selectively act on cells co-expressing two antigens

**DOI:** 10.1038/s41587-022-01384-1

**Published:** 2022-07-25

**Authors:** Simone C. Oostindie, Derek A. Rinaldi, Gijs G. Zom, Michael J. Wester, Desiree Paulet, Kusai Al-Tamimi, Els van der Meijden, Jennifer R. Scheick, Tessa Wilpshaar, Bart de Jong, Marloes Hoff-van den Broek, Rachel M. Grattan, Janita J. Oosterhoff, Julie Vignau, Sandra Verploegen, Peter Boross, Frank J. Beurskens, Diane S. Lidke, Janine Schuurman, Rob N. de Jong

**Affiliations:** 1 Genmab, Utrecht, the Netherlands; 2 Department of Immunology, Leiden University Medical Center, Leiden, the Netherlands; 3 Department of Pathology, University of New Mexico School of Medicine, Albuquerque, NM USA; 4 Department of Physics and Astronomy, University of New Mexico, Albuquerque, NM USA; 5 Comprehensive Cancer Center, University of New Mexico Health Sciences Center, Albuquerque, NM USA

**Keywords:** Antibody therapy, Protein design, Drug development

## Abstract

The use of therapeutic monoclonal antibodies is constrained because single antigen targets often do not provide sufficient selectivity to distinguish diseased from healthy tissues. We present HexElect^®^, an approach to enhance the functional selectivity of therapeutic antibodies by making their activity dependent on clustering after binding to two different antigens expressed on the same target cell. lmmunoglobulin G (lgG)-mediated clustering of membrane receptors naturally occurs on cell surfaces to trigger complement- or cell-mediated effector functions or to initiate intracellular signaling. We engineer the Fc domains of two different lgG antibodies to suppress their individual homo-oligomerization while promoting their pairwise hetero-oligomerization after binding co-expressed antigens. We show that recruitment of complement component C1q to these hetero-oligomers leads to clustering-dependent activation of effector functions such as complement mediated killing of target cells or activation of cell surface receptors. HexElect allows selective antibody activity on target cells expressing unique, potentially unexplored combinations of surface antigens.

## Abstract (web summary)

Antibody pairs are made to act selectively on cells co-expressing two targets by engineering their Fc domains.

## Main

Monoclonal antibodies (mAbs) have reshaped drug development due to their highly targeted nature and their ability to activate specific immune effector molecules and cells. Superior effector functions have been engineered into mAbs to enhance therapeutic efficacy^1^. Complement- or cell-mediated effector functions can be triggered by antigen-dependent immunoglobulin G (IgG) oligomerization into ordered hexameric complexes on the cell surface through noncovalent Fc–Fc interactions^2^. These ordered hexamers provide natural high-avidity binding sites for complement complex C1, leading to activation of the classical complement pathway, or to receptor clustering and outside-in signaling^3–6^. IgG hexamerization and effector function activation can be enhanced by single amino acid point mutations in the Fc domain, such as E430G, that promote interactions between Fc domains of cell-bound IgG at physiologically relevant concentrations^2,7^, and can promote solution interactions at highly elevated concentrations in specific formulations^8^. Hexamerization-enhanced antibody variants increased complement-dependent cytotoxicity (CDC) of B cells from patients with chronic lymphocytic leukemia (CLL) and were able to facilitate enhanced clustering and activation of members of the tumor necrosis factor receptor superfamily^3,6,7,9^.

The target space for therapeutic mAbs based on natural IgG backbones is largely limited to those targets that are selectively expressed on diseased cells. Hence, there may be advantages to therapeutic antibody technologies that enable the safe and effective targeting of antigens that currently cause undesirable toxicity on healthy cells, or enable insufficient potency on diseased cells. We recently reported that mAbs targeting different membrane receptors can hetero-oligomerize into complexes on antigen binding, resulting in synergistic CDC of various tumor B cells^9^. Here, we describe a general approach to create IgG antibody pairs that only induce activation of oligomerization-dependent functions if both antibodies have bound the same target cell. Decoupling functional activation from individual target binding enables these IgG antibody pairs to act as Boolean logic AND gates that integrate two antibody-binding signals into a functional outcome only on cells or surfaces co-expressing both antibody targets.

## Results

### CD52/CD20 Ab mixtures broadly deplete hematological cells

**Fig. 1:**
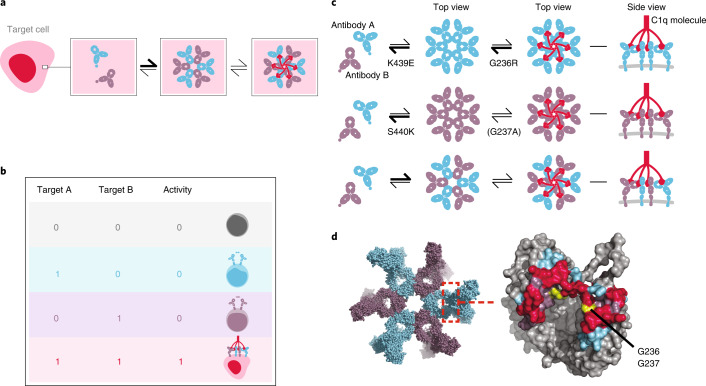
Design of Fc domain engineered IgG antibody pairs acting as Boolean logic AND gates. **a**, Schematic of antibody combinations that contain Fc domain modifications designed to induce hetero-hexamer formation only after cell surface target binding, aiming to recruit complement component C1q and induce effector function activation. **b**, Mutually dependent antibody pairs may act as Bio-Logic AND gates by integrating two antibody-binding input signals into a functional output exclusively on cells or surfaces that co-express both antibody targets. **c**, Summary of coupled biochemical equilibria illustrating how IgG1-hexamer-C1q avidity can be tuned using K439E/S440K and G236R/G237A mutations to favor hetero-hexamerization over homo-hexamerization. **d**, Left, an overview of an IgG1 antibody hetero-hexamer based on the IgG1-b12 crystal structure (1HZH)^42^. Right, detailed view of an Fc hinge domain indicating amino acid positions involved in C1q- (blue) and FcγR binding (purple). The largely shared C1q- and FcγR binding interface (magenta) highlights preferred amino acid positions that differentially modulate C1q and FcγR binding (yellow).

Previously, we showed that mutating amino acid positions Lys^439^ and Ser^440^ at the Fc–Fc interface into glutamate (K439E) and lysine (S440K), respectively, creates a pair of antibody mutants that show reduced homo-hexamerization, but efficiently form hetero-hexamers in mixtures containing both mutants (Supplementary Fig. 1)^2,4,9^. Here, we investigated whether these mutations can be used to create antibody pairs targeting two different antigens that form hetero-hexameric complexes only when bound to the same target cell, enabling them to act as the biological equivalent of a logic AND gate. These mutually dependent antibody pairs aim to activate effector functions only on cells expressing a combination of two targets A AND B, while preventing activation on cells expressing only target A OR target B (Fig. 1a,b).

To engineer antibody pairs that enable mutually dependent activation, we chose a model system composed of antibodies targeting the abundantly expressed and well-characterized surface antigens CD52 and CD20. We generated IgG1 antibodies with hexamerization-enhancing mutation E430G that target CD52 (IgG1-Campath-E430G), commonly expressed on T cells and B cells^10^, and CD20 (IgG1-11B8-E430G), primarily expressed on B cells^11^. A mixture of IgG1-Campath-E430G and IgG1-11B8-E430G efficiently killed Wien-133 lymphoma B cells and depleted both B cells and T cells in human whole blood (Supplementary Fig. 2). Introducing mutations K439E and S440K to create antibody combination IgG1-Campath-E430G-K439E and IgG1-11B8-E430G-S440K was not sufficient to induce selective killing of only B cells expressing both CD52 and CD20. The killing of T cells by the antibody combination, despite T cells only expressing CD52, indicates that residual independent effector function activation might cause broad cell depletion. When tested individually, in the presence of a nonbinding control antibody (IgG1-b12) to keep IgG concentrations directly comparable, both single agents displayed substantial residual activity in whole blood (Supplementary Fig. 2). Analysis of individual antibody effector functions in B-lymphoma cell lines demonstrated that IgG1-Campath-E430G-K439E still induced residual complement activation while IgG1-Campath-K439E did not (Supplementary Fig. 2), indicating that complement regulation was compromised by promoting Fc–Fc interactions. Furthermore, both single components, IgG1-Campath-E430G-K439E and IgG1-11B8-E430G-S440K, still activated Fc gamma receptor (FcγR) IIa- and IIIa-dependent signaling in cell-based reporter assays (Supplementary Fig. 2), suggesting that their activation was not strictly dependent on antibody oligomerization. Thus, this pair of antibody variants did not act in a fully mutually dependent fashion, since selective activation of effector functions by K439E and S440K mutant antibody pairs was compromised both by residual antibody clustering on single target cells, and by FcγR activity that was not strictly dependent on antibody oligomerization.

### IgG1 effector function modulation by G236/G237 mutations

**Fig. 2:**
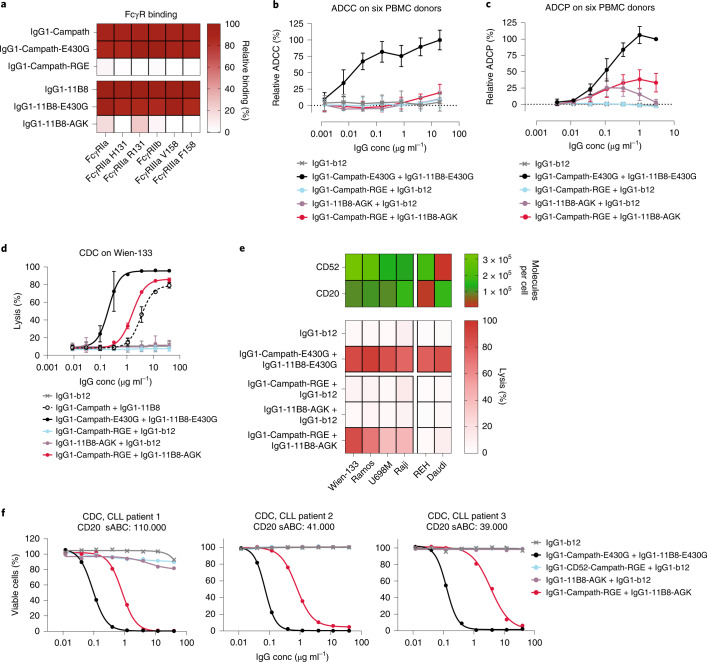
Clustering-dependent effector function activation by differential modulation of effector binding through G236/G237 mutations. **a**, Binding of 20 µg ml^−1^ IgG1-Campath and IgG1-11B8 antibody variants to FcγRs (ELISA). Data were normalized to IgG1-b12 (0% binding) and wild-type IgG1-Campath or IgG1-11B8 (100% binding) and presented as the mean of ﻿﻿*n* = 3 independent experiments. **b**,**c**, ADCC (**b**) or ADCP (**c**) by IgG1-Campath and IgG1-11B8 antibody variants using Wien-133 target cells (**b**) and PBMC effector cells (E:T 1:100), or Calcein AM-labeled Raji target cells (**c**) and CD11b^+^ h-MDM effector cells (E:T 2:1). Data were normalized to IgG1-b12 (0% ADCC/ADCP) and a mixture of IgG1-Campath-E430G and IgG1-11B8-E430G (100% ADCC/ADCP). Mean and s.d. over *n* = 6 donors are shown. **d**, CDC of Wien-133 cells by IgG1-Campath and IgG1-11B8 antibody variants. Mean and s.d. over *n* = 3 independent experiments are shown. ﻿**b**,**c**,**d**, Statistical analysis of AUC values is described in Supplementary Table 3. **e**, CDC (lower panel) induced by IgG1-Campath and IgG1-11B8 antibody variants (40 µg ml^−1^ final concentration) in different tumor B cell lines expressing various levels of CD52 and CD20 quantified by the number of antibody molecules bound per cell (top panel). Data shown are mean values over *n* = 4 independent experiments. **f**, Dose-response CDC in PBMCs derived from three patients with CLL opsonized with serial dilutions of IgG1-Campath and IgG1-11B8 mutant antibody variants and ranked according to CD20 expression levels. sABC, specific antibody-binding capacity.

To increase the selectivity of effector function activation by K439E and S440K mutant antibody pairs, we differentially modulated the binding to FcγR and C1q effector molecules (Fig. 1c). IgG (hetero-) hexamer abundance and stability are the product of both Fc–Fc and Fc–C1q interactions^2,5,12^ and the binding sites for C1q and FcγRs show substantial overlap^13–15^ (Fig. 1d). We examined several amino acids at the shared IgG binding interface that differentially modulated FcγR and C1q binding affinity. Mutations G236R and G237A were selected to simultaneously maximize the therapeutic index between individual and mutually dependent C1q recruitment, and to suppress or eliminate FcγR-mediated effector functions such as antibody dependent cellular cytotoxicity (ADCC) and antibody dependent cellular phagocytosis (ADCP) (Supplementary Fig. 3 and Fig. 1c,d). Oligomerization-independent FcγR binding and activation was strongly suppressed by introducing G236R in IgG1-Campath-E430G-K439E (IgG1-Campath-RGE) and G237A in IgG1-11B8-E430G-S440K (IgG1-11B8-AGK), both individually and in combination (Fig. 2a and Supplementary Fig. 3). In a functional assay, ADCC was not detected using either the single antibody components or the antibody combination (Fig. 2b). By contrast, low level ADCP activity was still detectable, which may be attributed to residual binding to FcγRI and FcγRIIa by IgG1-11B8-AGK (Fig. 2c and Supplementary Fig. 7). Tuning of the C1q binding affinity using G236R eliminated the single agent CDC activity of IgG1-Campath-RGE on Wien-133 tumor B cells. Mixed IgG1-Campath-RGE and IgG1-11B8-AGK recovered CDC activity comparable to a mixture of wild-type IgG1-Campath and IgG1-11B8 without E430G mutations (Fig. 2d), indicating that complement activation was strictly mutually dependent. These observations were corroborated by analysis of C1q binding (Supplementary Fig. 3). Nonequimolar target expression, and the asymmetric reduction of C1q binding affinity imposed by mutations G236R and G237A, may explain why mixed, mutually dependent IgG1-Campath-RGE and IgG1-11B8-AGK components showed C1q binding and CDC comparable to a mix of unmodified IgG1 antibodies, but did not recover the maximal potency of mixed, independently acting antibodies with only E430G hexamerization-enhancing mutations.

To assess whether cytotoxicity of the IgG1-Campath-RGE and IgG1-11B8-AGK antibody combination is restricted to cells co-expressing CD52 and CD20, we analyzed the CDC activity of individual and mixed antibody components on six tumor B cell lines with variable CD52 and CD20 expression levels. All six cell lines were sensitive to CDC by a combination of IgG1-Campath-E430G and IgG1-11B8-E430G. Tested as single components, neither IgG1-Campath-RGE nor IgG1-11B8-AGK induced CDC on any of the cell lines tested (Fig. 2e). In contrast, the combination of IgG1-Campath-RGE and IgG1-11B8-AGK induced mutually dependent CDC on CD52/CD20 double positive cell lines only, in which the maximal lysis was dependent on the relative expression of both targets. Furthermore, the potency of the mutually dependent CD52 and CD20 antibody combination was tested on B cells derived from peripheral blood mononuclear cells (PBMCs) of patients with CLL ex vivo. The IgG1-Campath-RGE and IgG1-11B8-AGK antibody combination, but not the individual antibody components, induced almost complete lysis of B cells derived from three different patients with CLL (Fig. 2f and Supplementary Table 9).

### Selective depletion of B cells in heterogeneous mixtures

**Fig. 3:**
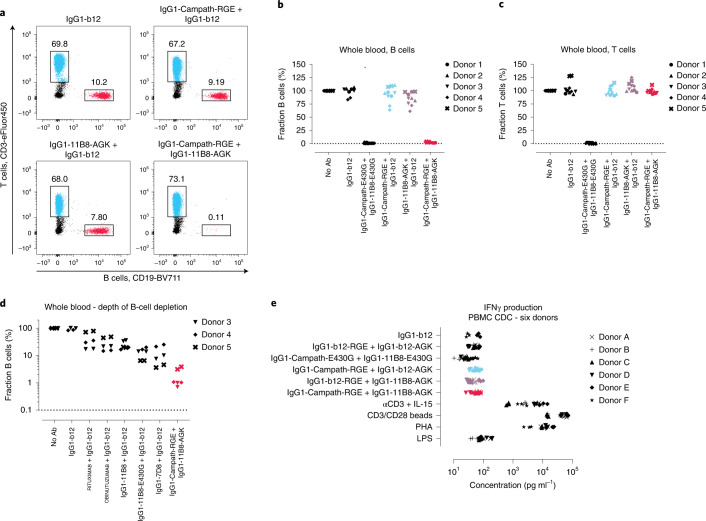
A mutually dependent CD52 and CD20 antibody combination induces selective depletion of B cells. **a**–**d**, Cytotoxicity induced by IgG1-Campath and IgG1-11B8 antibody variants (10 µg ml^−1^ final concentration) incubated for 18 h in healthy human whole blood, as analyzed by flow cytometry. PBMC viability after 18 h exceeded 90% for all nontreated control samples. **a**, The fraction (%) of B or T cells remaining within the lymphocyte population (CD66b^−^) is shown for one representative donor. **b**,**c**, Summary of cytotoxicity results relative to a nontreated (no antibody) control sample for five donors containing variable levels of B (**b**) and T cells (**c**). **d**, B cell depletion induced by mutually dependent IgG1-Campath and IgG1-11B8 antibody combinations compared to that of different existing CD20 antibody molecules, shown for three donors relative to a nontreated (no antibody) control sample. **e**, PBMCs isolated from the blood of six healthy donors were incubated for 20–24 h in the presence of 10 µg ml^−1^ of each antibody mixture and 20% (V/V) complement-competent NHS. Supernatant cytokine levels were analyzed by MSD QuickPlex Cytokine analysis. Interferon gamma levels shown were measured for six donors, in three individually incubated samples per donor, collected in two experiments. A mixture of anti-CD3 and IL-15, anti-CD3 and anti-CD28 antibodies immobilized on beads, phytohemagglutin (PHA) and lipopolysaccharide (LPS) served as positive control stimuli of different cytokine responses.

We evaluated B and T cell depletion by the mutually dependent anti-CD52 IgG1-Campath-RGE and anti-CD20 IgG1-11B8-AGK antibody combination in human whole blood. Indeed, this combination induced selective depletion of CD19^+^CD52^+^CD20^+^ B cells in human whole blood derived from five healthy donors, while the CD3^+^CD52^+^ T cell population lacking CD20 remained unaffected (Fig. 3a–c and Supplementary Fig. 7). IgG1-Campath-RGE or IgG1-11B8-AGK did not induce depletion of B or T cells independently, demonstrating that CDC was dependent on binding of both components to antigens co-expressed on the same target cell. Compared to CD20 antibodies known to deplete B cells in human whole blood^16–18^, the mutually dependent IgG1-Campath-RGE and IgG1-11B8-AGK antibody combination induced efficient depletion of B cells in human whole blood derived from three healthy donors (Fig. 3d).

To examine whether binding of mutually dependent antibodies induced cellular activation, we incubated healthy donor PBMCs with IgG1-Campath-RGE and IgG1-11B8-AGK mixtures and measured cytokine levels and cell surface markers associated with immune cell stimulation. Both independently and mixed, opsonization with these antibodies induced IFNγ levels comparable to isotype controls (Fig. 3e). IL-2, TNFα and IL-6 levels were also comparable to isotype controls under these conditions; induction of T cell activation markers CD25 or CD69 was not detected (Supplementary Fig. 3). In contrast, these cytokines and markers were induced by positive control stimuli such as anti-CD3/anti-CD28 beads, anti-CD3 + IL-15, phytohemagglutin and/or lipopolysaccharide. Mixed IgG1-Campath-E430G + IgG1-11B8-E430G reduced IFNγ and TNFα levels, likely explained by the depletion of T cells that can contribute to their production.

### Cell surface proximity of CD52/CD20 antibody combinations

**Fig. 4:**
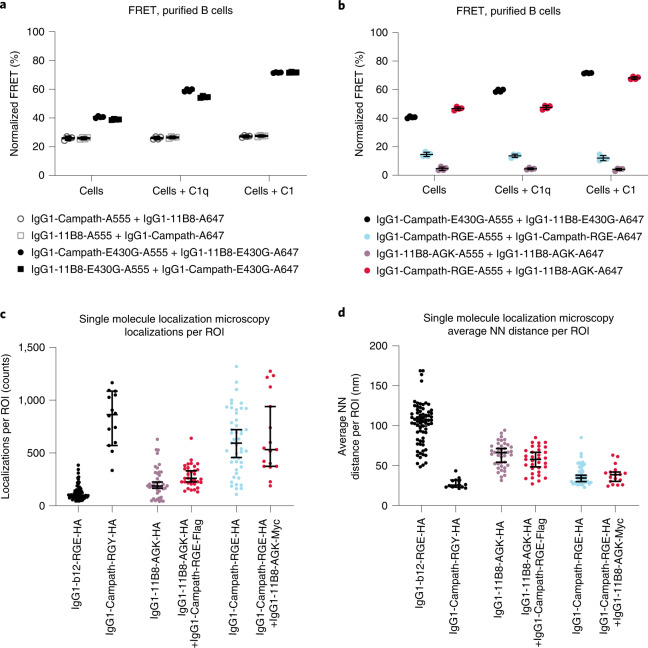
Cell surface proximity of CD52 and CD20 antibody combinations. **a**,**b**, FRET analysis to detect the molecular proximity of (**a**) wild-type and E430G mutant IgG1-Campath or IgG1-11B8 antibody combinations or (**b**) IgG1-Campath and IgG1-11B8 mutually dependent antibody combinations. Purified healthy donor B cells were opsonized with 10 µg ml^−1^ A555-conjugated- and 10 µg ml^−1^ A647-conjugated antibody variants in the presence or absence of purified human C1q (2.5 µg ml^−1^) or C1 (2.42 µg ml^−1^) and FRET was calculated from normalized MFI values as determined by flow cytometry. Data shown are mean and s.d. of four measurements collected in *n* = 2 independent experiments. **c**,**d**, Super-resolution image analysis of lateral protein organization. Wien-133 cells were opsonized with 5.0 µg ml^−1^ of the indicated antibody mixtures in the presence of 2.5 µg ml^−1^ C1q, followed by fixation and labeling with anti-HA-AF647 antibody. Regions of interest (ROI) were selected from multiple cells acquired over at least two independent experiments, with the number of cells ranging from 7 to 23 and with *n* being the number of analyzed ROI ranging from 14 to 69 depending on the sample (Supplementary Table 4). Error bars indicate median and 95% confidence interval. Statistical comparisons between distributions are described in Supplementary Table 4. **c**, Number of localizations per ROI. **d**, Average nearest neighbor (NN) distance per ROI.

The molecular proximity of antibody combinations targeting CD52 and CD20 on B cells was examined using Förster resonance energy transfer (FRET) analysis. Purified human B cells from healthy donors were opsonized with fluorescently labeled CD52 and CD20 antibody variants added either individually or in combination. While wild-type CD52 and CD20 antibody combinations elicited limited proximity-induced FRET, introduction of hexamerization-enhancing mutation E430G increased FRET efficiency (Fig. 4a). Adding purified C1q or C1 enhanced the FRET interaction between CD52 and CD20 antibodies only if they contained mutation E430G, suggesting that Fc–Fc interactions and C1q or C1 recruitment cooperatively promoted close antibody proximity. The mutually dependent CD52 and CD20 antibody combination IgG1-Campath-RGE + IgG1-11B8-AGK induced a FRET efficiency similar to combination IgG1-Campath-E430G + IgG1-11B8-E430G, whereas FRET was substantially reduced for individual components (Fig. 4b).

Despite its high sensitivity for detecting protein interactions, FRET does not provide details on the lateral organization of proteins on the cell membrane. To assess antibody nanoscale organization, we performed direct stochastic optical reconstruction microscopy (dSTORM). This single molecule localization-based technique provides roughly 15 nm resolution and allows for the detection of small changes in protein distribution or proximity^19,20^. Direct fluorescent labeling of Campath or 11B8 antibodies at the levels required for dSTORM negatively affected their CDC potency, but hemagglutinin (HA), myc or Flag peptides introduced at the light chain C terminus had minimal impact (Supplementary Fig. 4). Cells incubated with HA-tagged antibodies were fixed and labeled for dSTORM imaging using an AlexaFluor647 (AF647)-tagged anti-HA antibody (Supplementary Fig. 5).﻿ Nonspecific binding of the anti-HA-A647 antibody was detected in an IgG1-b12-RGE-HA isotype control sample at levels lower than for CD20- or CD52-targeted antibodies. Consistent with CD20 and CD52 expression on Wien-133 cells, IgG1-11B8-AGK-HA showed fewer localizations per region of interest (ROI) than IgG1-Campath-RGE-HA (Fig. 4c). Control variant E430G-E345R-S440Y (RGY) induces IgG hexamerization in solution^2,12^, and labeling of variant IgG1-Campath-RGY-HA was similar to that of IgG1-Campath-RGE-HA. Notably, IgG1-Campath-RGE-HA and IgG1-11B8-AGK-HA showed similar localizations per ROI in the absence or presence of their unlabeled complementary components IgG1-11B8-AGK-myc and IgG1-Campath-RGE-Flag, respectively. To quantify the protein distribution on the cell surface, we calculated the nearest neighbor distances between antibodies (Fig. 4d and Supplementary Fig. 5). We found that the average nearest neighbor distance inversely correlated with the amount of labeling for each variant, that is, the low nonspecific labeling of IgG1-b12-RGE-HA is further from its nearest neighbor than the more densely labeled IgG1-Campath-RGE, with IgG1-11B8-AGK falling in between. Prehexamerizing IgG1-Campath-RGY-LHA induced the lowest average nearest neighbor distance per ROI. Contrary to our expectation, the distribution of IgG1-11B8-AGK-LHA was not changed on addition of its unlabeled partner, IgG1-Campath-RGE-Flag and, conversely, the distribution of IgG1-Campath-RGE-LHA was not changed by addition of IgG1-11B8-AGK-myc. Since individual antibody components and mutually dependent combinations showed a similar number of localizations and nearest neighbor distances, the engineered Fc–Fc interactions did not appear to induce large-scale target reorganization or aggregation on the cell membrane.

### Mutually dependent Abs selectively deplete B cells ﻿in vivo

**Fig. 5:**
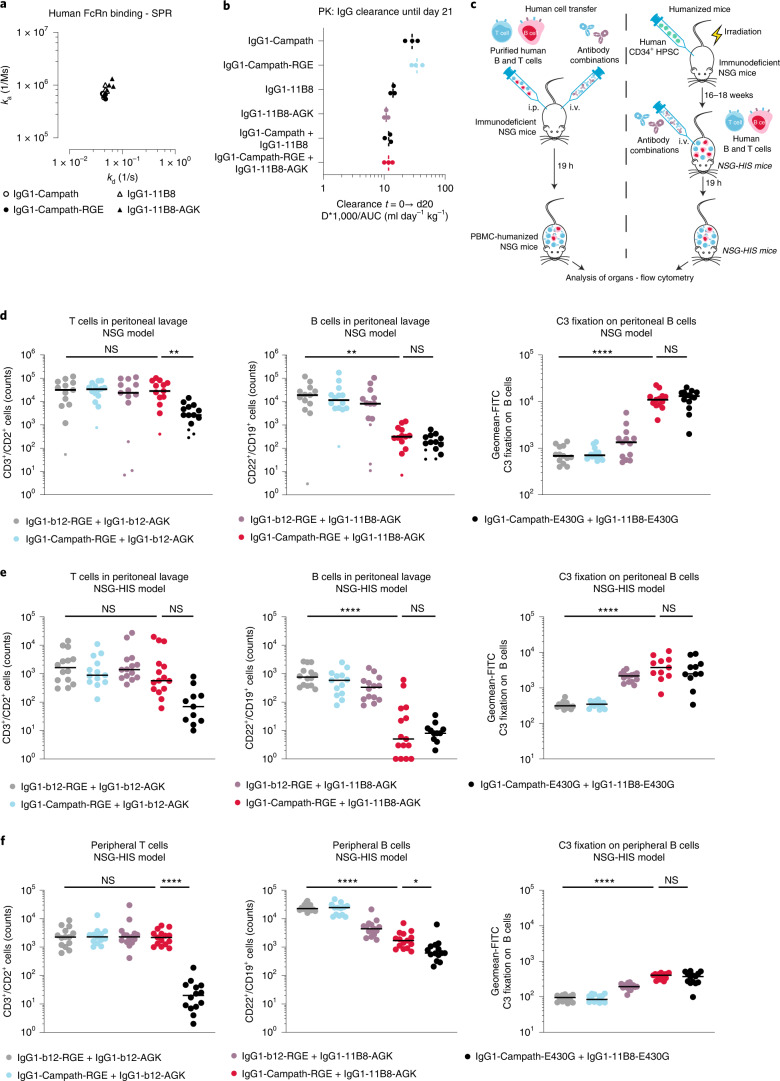
A mutually dependent CD52/CD20 antibody combination induces selective depletion of B cells ﻿in vivo. **a**, Kinetics of binding by a dose-range of IgG variants to human FcRn immobilized on sensor chips, determined by Surface Plasmon Resonance at pH 6.0; binding at pH 7.4 was undetectable. Individually determined *k*_a_ and *k*_d_ rate constants (determined in *n* = 3 independent experiments) are plotted, see Supplementary Table 5 for statistical comparisons. **b**, Pharmacokinetic analysis of IgG1-Campath and IgG1-11B8 antibody variants in SCID mice (*n* = 3 mice per group). Clearance of a single antibody dose (500 μg) was monitored for 3 weeks and is expressed as dose (*D*) × 1,000/AUC. See Supplementary Table 6 for statistical comparisons. **c**, Schematic summary of ﻿in vivo mouse model study designs as examined in **d**–**f**. Left, NSG human cell transfer model. Right, humanized NSG-HIS model. **d**, Depletion of transferred human cells: human T and B cells (8 million per mouse) were injected intraperitoneally (i.p.) in NSG mice and mice were treated with the indicated antibody combinations intravenously (i.v.) (at a combined dose of 1 mg kg^−1^). Cells were recovered by peritoneal lavage 19 h after the treatment and analyzed by flow cytometry. Graphs summarize flow cytometry analyses of the count of human CD3^+^/CD2^+^ T cells (left), count of human CD22^+^/CD19^+^ B cells (center) and mean fluorescence intensity of complement C3 fragment deposition on human CD22^+^/CD19^+^ B cells detected by anti-C3-FITC conjugate (right). Small symbols indicate mice from which low numbers of total human CD45^+^ cells were recovered. No mice were excluded based on cell counts, but lipid contaminations precluded analysis of some samples. **e**,**f**, Depletion of human cells in humanized NSG mice (NSG-HIS). A cohort of NSG-HIS mice with circulating human immune cells generated from three different healthy human donors were randomized into different treatment groups based on donors and human CD45^+^ cell counts. Mice were injected intravenously with antibody combinations (at a combined dose of 0.5 mg kg^−1^). After 19 h of treatment, human T and B cells in peritoneal lavage (**e**) and blood (**f**) were analyzed by flow cytometry using the same markers described above. In both depletion models, mice were pretreated with 8 mg human IVIg 1 day before treatment to restore circulating immunoglobulin levels. **d**–**f**, Black lines indicate median values; statistical differences between groups are indicated by *P < 0.05, **P < 0.01, ***P < 0.001 and ****P < 0.0001; *n* = 13 to 14 mice per group (**d**), resp. 11 to 15 mice per group (**e**); see Supplementary Table 7 for detailed statistical comparisons.

Preserving regular human FcRn binding and pharmacokinetics is important for potential therapeutic applications of engineered antibodies. IgG1-Campath-RGE and IgG1-11B8-AGK both displayed human FcRn binding similar to their respective wild-type antibodies at pH 6.0 (Fig. 5a and Supplementary Fig. 4). Kinetic parameters could not be determined at pH 7.4, because wild-type and variant antibodies showed undetectable binding. The pharmacokinetic profiles of IgG1-Campath and IgG1-11B8 antibodies were analyzed for three weeks in the absence of target binding in tumor-free C.B-17 severe combined immunodeficiency (SCID) mice to avoid antidrug antibody responses (Fig. 5b). Wild-type IgG1-Campath displayed slightly faster clearance than wild-type IgG1-11B8. Both single IgG1-Campath-RGE and IgG1-11B8-AGK antibody components showed clearance rates comparable to those of wild-type IgG1-Campath and IgG1-11B8, respectively. Mixed IgG1-Campath-RGE and IgG1-11B8-AGK displayed a clearance rate similar to that of mixed wild-type IgG1-Campath and IgG1-11B8 and that of the slower clearing IgG1-11B8.

The ﻿in vivo depletion of human B and T cells by different antibody combinations was tested in two different mouse models (Fig. 5c). First, purified human B and T cells from different healthy donors were injected intraperitoneally into severely immuno-deficient NOD-SCID-gamma (NSG) (NOD/SCID/IL2Rγ-chain null) mice, which lack B, T and Natural Killer cells, followed by intravenous administration of antibody mixtures. Mice were pretreated with IVIg (Octagam) to provide competing IgG levels otherwise absent from B cell-deficient mice. On the following day, remaining B and T cells were recovered by peritoneal lavage (Fig. 5d and Supplementary Figs. 6 and 7). Similar numbers of B and T cells were recovered after exposure to mixtures of nonbinding IgG1-b12-RGE + IgG1-b12-AGK, IgG1-Campath-RGE + IgG1-b12-AGK or IgG1-11B8-AGK + IgG1-b12-RGE. Positive control antibody mixture IgG1-Campath-E430G + IgG1-11B8-E430G induced depletion of both B and T cells. Antibody combination IgG1-Campath-RGE + IgG1-11B8-AGK induced selective depletion of B cells, but not T cells (Fig. 5d), consistent with whole blood assays ﻿in vitro (Fig. 3a–c). Mixed IgG1-Campath-RGE + IgG1-11B8-AGK increased the deposition of complement C3 fragments only on remaining B cells, while the positive control mixture induced complement deposition on both B and T cells (Fig. 5d and Supplementary Fig. 6).

In the first mouse model, the transferred human cells do not migrate out of the peritoneum within the time span of the study, indicating that circulatory cell dynamics are not well represented. We therefore corroborated these findings in a second ﻿in vivo model using NSG-HIS (humanized immune system) mice containing circulating T and B cells developed from human donor hematopoietic stem cells. Different antibody combinations were administered intravenously and human B and T cells were recovered 24 h later by peritoneal lavage, isolated from blood and analyzed by flow cytometry (Fig. 5e,f and Supplementary Fig. 7). Positive control mixture IgG1-Campath-E430G + IgG1-11B8-E430G induced depletion of, and C3 fragment deposition on, both B and T cells. In contrast, IgG1-Campath-RGE + IgG1-11B8-AGK selectively depleted and induced C3 deposition on B cells, but not T cells (Supplementary Fig. 6). IgG1-Campath-RGE mixed with IgG1-b12-AGK maintained strict complement regulation. Of note, control antibody mixture IgG1-11B8-AGK + IgG1-b12-RGE induced C3 fragment deposition on human B cells and depletion of peripheral B cells (Fig. 5e,f), although less efficiently than IgG1-Campath-RGE + IgG1-11B8-AGK. In summary, IgG1-Campath-RGE + IgG1-11B8-AGK induced selective B cell depletion ﻿in vivo, while FcRn binding and clearance were not affected by the presence of triple mutations.

### Generalization of mutually dependent antibody combinations

**Fig. 6:**
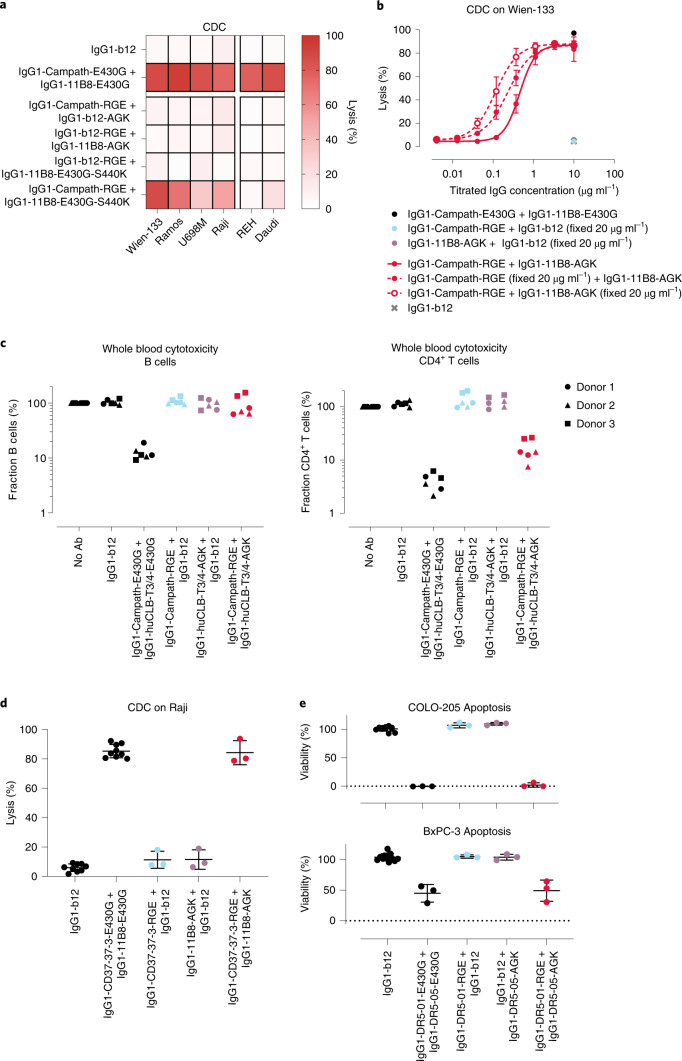
Mutually dependent antibody combinations are applicable to different cell surface antigen combinations. **a**, CDC induced by IgG1-Campath and IgG1-11B8 antibody variants (40 µg ml^−1^ final concentration) on different tumor B cell lines. Data shown are mean values of at least three independent experiments. **b**, Dose-response CDC on Wien-133 cells opsonized with nonequimolar mixtures of mutually dependent IgG1-Campath and IgG1-11B8 antibody combinations. Titrated antibodies were mixed with antibodies at a fixed (10 µg ml^−1^) concentration. Mean and s.d. over *n* = 3 independent experiments are shown. **b**,**d**, See Supplementary Table 3 for statistical analysis of AUC values. **c**, Cytotoxicity induced by anti-CD3 mAb IgG1-huCLB-T3/4 and IgG1-Campath antibody variants (10 µg ml^−1^ final concentration) incubated for 45 min in healthy human whole blood, as analyzed by flow cytometry. The fraction (%) of B cells and CD4^+^ T cells remaining within the lymphocyte population (CD66b^−^) is shown for three individual donors relative to a nontreated (no antibody) control sample. **d**, CDC on Raji cells opsonized with 40 µg ml^−1^ anti-CD37 mAb IgG1-37.3 and IgG1-11B8 antibody variants. Mean and s.d. over *n* = 3 independent experiments are shown. **e**, COLO-205 and BxPC-3 cells were incubated with 20 µg ml^−1^ anti-DR5 IgG1-DR5-01 and IgG1-DR5-05 antibody variants in the presence of 2.5 µg ml^−1^ purified human C1q and cell viability (%) was measured after 72 h. Mean and s.d. over *n* = 3 independent experiments are shown. **d**,**e**, See Supplementary Table 3 for statistical analysis.

One mechanism potentially contributing to the observed ﻿in vivo activity of antibody mixture IgG1-11B8-AGK + IgG1-b12-RGE could be the recruitment of nonbinding antibodies from solution by cell surface-bound antibodies if both contain complementary mutations. On a panel of six human tumor B cell lines in normal human serum (NHS), CDC activity could not be detected for IgG1-Campath-RGE + IgG1-b12-AGK or for IgG1-11B8-AGK + IgG1-b12-RGE (Fig. 6a). This indicates that, under these conditions, the recruitment of circulating antibodies did not meaningfully contribute to tumor cell killing by mutually dependent antibody combinations.

For tumor targeting applications, it could be beneficial to selectively permit ADCC activity for a tumor-specific antibody component (for example, CD20), when used in combination with a component that targets a more broadly expressed antigen (for example, CD52). We therefore analyzed how preserving IgG1-11B8-E430G-S440K FcγR binding (Supplementary Fig. 2) affected the CDC activity after mixing with IgG1-Campath-RGE (Fig. 6b). This functionally asymmetric antibody combination retained selective activation of CDC only on CD52^+^CD20^+^ cell lines, while recruiting more C1q than the FcγR-suppressed antibody combination (Fig. 6b and Supplementary Fig. 3). These results indicate that effector functions of such antibody combinations can be adapted to the target expression profiles.

Exchanging the Fc backbones applied in IgG1-Campath and IgG1-11B8 was analyzed in recalcified whole blood assays (Supplementary Figs. 6 and 7). While IgG1-Campath-RGE retained selective depletion of B cells, IgG1-Campath-AGK induced some dose-dependent depletion of T cells, albeit less efficiently than the positive control mixture. This observation is consistent with the lower CDC potency of IgG1-Campath-E430G-G236R than IgG1-Campath-E430G-G237A (Supplementary Fig. 3), suggesting that reducing C1q affinity in the IgG1-Campath-RGE component contributed to increased selectivity. IgG1-11B8-AGK and -RGE were both inactive in absence of a complementary component.

We also evaluated how mixing mutually dependent antibody components at nonequimolar concentrations affected CDC potency. At saturating concentrations of either IgG1-Campath-RGE or IgG1-11B8-AGK, a titration of the second antibody component recovered essentially identical CDC activity compared to an equimolar mixture, indicating that specificity and potency was preserved for a range of different mixture compositions (Fig. 6b).

Potential applications of mutually dependent antibody combinations composed of one antibody with RGE mutations and one with AGK mutations were further explored for different target combinations. We tested if combining IgG1-Campath-RGE with CD3-directed IgG1-huCLB3/4-AGK could induce selective T cell depletion in recalcified human whole blood. Indeed, this antibody combination induced selective depletion of CD4^+^CD52^+^ T cells, while sparing CD52^+^ B cells (Fig. 6c). T cell depletion was strictly mutually dependent, as neither individual antibody induced T or B cell depletion. Furthermore, a combination of antibodies targeting CD37 (IgG1-CD37.3-RGE) and CD20 (IgG1-11B8-AGK) induced potent and mutually dependent CDC on a CD37^+^CD20^+^ tumor cell line (Fig. 6d).

We have shown that mutually dependent antibody combinations could regulate oligomerization-dependent complement activation. Binding of hexamerization-enhanced antibodies to tumor necrosis factor receptor superfamily members such as death receptor 5 (DR5) and OX40, which are activated via higher order receptor clustering, were also reported to enhance agonistic signaling and tumor cell death (Supplementary Fig. 6)^3,6^. Hence, we evaluated whether a mutually dependent antibody combination targeting nonoverlapping epitopes on DR5 (IgG1-DR5-01-RGE and IgG1-DR5-05-AGK) could achieve agonistic activation. Indeed, while both single antibodies were silent, IgG1-DR5-01-RGE + IgG1-DR5-05-AGK potently induced apoptosis in COLO-205 and Bx-PC3 solid tumor cell lines with high and intermediate sensitivity to DR5-targeting, respectively (Fig. 6e)^3^. In contrast, dual epitope targeting by the two wild-type antibodies showed limited to no efficacy (Supplementary Fig. 6). Collectively, these results illustrate that mutually dependent antibody combinations can be designed to target different cell surface antigen combinations, and to elicit potent, specific complement activation or agonistic activation of target signaling.

## Discussion

Activation of antibody-mediated effector functions to eliminate pathogens or destroy tumor cells primarily requires avid antibody binding to cognate antigens on the target cell. Potent effector activity by antibody-based therapies, however, may lead to undesired on- or off-target toxicity^21^. Here, we describe an AND-gated approach to decouple effector function activation by antibody combinations from individual antibody-binding events. Both antibodies may target different membrane receptors and bind various cellular subtypes individually, but specific Fc-region point mutations modulate Fc–Fc, C1q and FcγR interactions to make IgG hetero-oligomerization and functional activation stringently dependent on the presence of both antibodies^2,7,9,22^. This AND-gated approach was applicable to the regulation of complement activation and agonistic signaling, and to multiple target combinations present on both hematologic and solid tumor cells. Asymmetric silencing of FcγR-mediated effector functions within antibody combinations was possible, enabling design variations that mitigate or leverage on-target toxicity depending on target expression profiles and desired effects.

In some assays, the G237A mutation appeared to only partially suppress FcγR binding and ADCP activity consistent with recent findings^23^, which may limit its application when targeting some broadly and highly expressed antigens, although selectivity was preserved in whole blood assays and in an NSG mouse model (Fig. 5). AGK and RGE variants of IgG1-11B8 maintained regulation on different human cell lines ﻿in vitro, but applied to IgG1-Campath, variant RGE displayed selectivity superior to AGK, consistent with G236R-induced C1q binding reduction (Fig. 1 and Supplementary Figs. 3 and 6). In an NSG-HIS model, IgG1-11B8-AGK induced partial depletion of and murine complement deposition on human B cells, particularly in the periphery. Human membrane complement regulatory proteins (mCRP) such as CD59 may regulate human complement more stringently than murine complement factors^24^.

On cells co-expressing both targets, mutually dependent antibody mixtures were generally less potent than combinations of independently acting, hexamerization-enhanced IgG1 antibodies and more potent than mixtures of unmodified, wild-type IgG1 antibodies. Nonequimolar or noncolocalizing target expression may allow antibodies bound to a locally less abundant target to functionally engage only an equimolar fraction of antibodies bound to the excess target if fully dependent on hetero-hexamerization. While FRET assays showed the complement-dependent proximity of antibody pairs on the cell surface, super-resolution microscopy demonstrated that this proximity is not the result of global target reorganization (Fig. 4). This suggests that subtle, local changes may be sufficient to support complement activation. The frequency at which hetero-hexamers or smaller hetero-oligomers may form and dissociate at the cell surface remains uncertain, and work to determine the exact stoichiometry of these complexes is ongoing. Previously, we could not detect C1q binding to IgG1 oligomers smaller than six, and showed that mutations K439E and S440K could impose the alternating assembly of hetero-hexamers^4,12^, suggesting that hetero-hexamers may be the oligomeric state required for IgG1-mediated recruitment of C1q to cells (Supplementary Fig. 3). The potency of mutually dependent antibody mixtures may also be affected by the reduced C1q affinity of component RGE in hetero-oligomeric assemblies (Supplementary Figs. 3 and 6). Nevertheless, a mutually dependent antibody mixture induced CDC activity rivaling that of known B cell depleting CD20 antibody molecules ﻿in vitro (Fig. 4e), and induced potent depletion of, and complement deposition on, B cells in mouse models (Fig. 5). NSG mice were reported to be defective in murine C5 (ref. ^25^), and it is uncertain whether C5 produced by human cells in these models may locally restore the terminal pathway of complement. We suspect that murine myeloid cells expressing complement receptors may contribute substantially to depletion of C3-opsonized cells in these models, but detailed mechanistic analyses will require additional research. In addition, to extend the present findings obtained with SCID mice, it could be desirable to analyze the pharmacokinetics of mutually dependent antibodies and mixtures in mice expressing human FcRn, and in mouse models that contain IgG competing for FcRn binding.

Potential therapeutic applications of mutually dependent antibody combinations require targets that co-localize and/or allow for antibody hetero-oligomerization after target binding. This process may be affected by target-specific restraints other than abundance, such as antigen size, density, mobility, epitope-membrane distance, surface distribution and the compatibility of epitopes with antibody hetero-oligomer formation^11,26–30^. The challenge of identifying target and epitope combinations that recover maximal potency seems reminiscent of the challenges inherent to covalent bispecific antibody design^31^. Covalent multi-specific designs often rely on careful affinity optimization to achieve selective binding to cells expressing target combinations in *cis*, further complicated by the heterogeneity of target expression and cell surface distribution. Noncovalent mutually dependent antibody mixtures may broaden the therapeutic combinatorial target space and simultaneously enhance the therapeutic index by decoupling individual antibody-binding events from in *cis* functional activation on preferred cell types, thereby enhancing on-target-cell and decreasing off-target-cell effects. We hypothesized that highly potent, ‘toxic’ activity of antibodies binding broadly expressed, nontumor-specific targets could be made dependent on the presence of antibodies directed against disease-specific targets. Herein, we consistently observed that mutually dependent antibody pairs retained maximal potency at doses exceeding target saturation, while avoiding the potency loss referred to as the hook or prozone effect caused by the self-competition observed for covalent bispecific designs^32^. This is explained by the strict dependence of antibody hexamerization and C1q binding on prior cell surface antigen binding, while monomeric antibodies in solution are incapable of C1q binding. Appropriate dosing might theoretically represent another challenge in the design of mutually dependent antibody combinations, as both components in the mixture might show different pharmacokinetics. Experiments with a wide range of nonequimolar antibody concentrations retained consistent potency, suggesting that nonequimolar mixtures could provide an option to address differences in clearance rates of the two components. Although a direct comparison of mutually dependent antibody combinations with covalent bispecific designs was beyond the scope of this study, future therapeutic index comparisons of these designs will be highly interesting.

Artificial logic gates, inspired by electronic engineering, have been applied in synthetic biology to control biological processes^33–35^. In human disease, Boolean logic gates are increasingly examined for their potential to enhance therapeutic selectivity, in particular for improving the specificity of natural, or chimeric antigen receptor (CAR) T cell recruitment^36–40^. In another approach, Boolean logic was applied to create auto-inhibited AND-gated antibodies that rely on a second, exogenous factor to activate binding^41^. The AND-gated approach we describe here solely decouples antibody binding from functional activation to achieve selectivity and is therefore not dependent on the environmental presence of secondary molecules such as enzymes or proteases. The mutually dependent antibody combinations described herein are minimally engineered, display regular pharmacokinetic properties and preserve regular manufacturability and developability characteristics. We therefore expect this approach to be broadly applicable to IgG Fc domain-based therapeutics, and to be generalizable to multiple applications including direct cell killing, agonistic activation and potentially other more complex Boolean logic gates. Furthermore, the methodology described herein is readily applicable to combinatorial, high throughput screening of large antibody panels for drug discovery. As for any therapeutic, potential new drug candidates based on AND-gated IgG backbones will require careful assessment of efficacy, safety and different manufacturing and dosing strategies.

In summary, we present an approach to generate mutually dependent antibody combinations (HexElect) that only allow for pairwise hetero-oligomerization and subsequent effector function activation after binding to two targets co-expressed on the cell surface. This decoupling of effector function activation from individual antibody-binding events provides a unique opportunity to enhance selectivity while maintaining potency, and may allow for the creation of a next generation of differentiated antibody therapeutics.

## Methods

### Antibodies

Rituximab (MabThera) and obinutuzumab (Gazyvaro) were obtained from the pharmacy (UMC Utrecht). All other antibodies were recombinantly produced at Genmab as described^3^. Design of mutually dependent antibody mixtures was based on sampling previously described mutations combined with modifications at the shared binding interface for C1q and FcγRs within the IgG1 Fc domain^2,7^. Details of the variant selection were described in patent WO 2019/211472, and will be published elsewhere. Mutations to enhance or inhibit Fc–Fc interactions and/or Fc–C1q binding interactions were introduced in expression vectors encoding the antibody heavy chain either using Quikchange technology (Agilent Technologies) or via gene synthesis (Thermo Fisher Scientific), at indicated positions numbered according to Eu nomenclature^43^. Peptide tags encoding HA (‘YPYDVPDYA’), cMyc (‘EQKLISEEDL’) or FLAG (‘DYKDDDDK’) affinity tags were inserted by gene synthesis after the C-terminal cysteine of the light chain. Quality control of recombinant antibodies was performed by different methods as described previously^7^: capillary electrophoresis–sodium dodecyl sulfate on the Labchip GXII (Caliper Life Sciences/PerkinElmer Hopkinton) under reducing and nonreducing conditions (>90% intact IgG, >95% HC þ LC under reducing conditions), electrospray ionization–time-of-flight–mass spectrometry (Waters) or Orbitrap (Thermo Fisher Scientific), and high-performance–size-exclusion chromatography (aggregate level <5%; Waters Alliance 2975 separation unit, Waters). The following IgG1 antibodies targeting human antigens were used: CD52 (P31358) mAb Campath^44^, CD20 (P11836) mAbs 11B8 and 7D8 (refs. ^45–47^), CD3 (P07766) mAb huCLB3/4 (ref. ^48^), CD37 (P11049) mAb IgG1-37.3 (ref. ^49^), DR5 (O14763) mAbs DR5-01 and DR5-05 (ref. ^3^). mAb IgG1-b12 targeting HIV-1 antigen gp120 (Q9IZE4) was used as a nonbinding isotype control^50^.

### Cells and reagents

Daudi (human B cell lymphoma), Raji (human B cell lymphoma), Ramos (human B cell lymphoma), COLO-205 (colorectal cancer) and BxPC-3 (pancreatic cancer) cell lines were obtained from the American Type Culture Collection (nos. CCL-23, CCL-86, CRL-1596, CCL-222 and CRL-1687, respectively). The human B lymphoma cell line U-698-M and the human B precursor leukemia cell line REH were obtained from the Deutsche Sammlung von Mikroorganismen und Zellkulturen (cell line numbers ACC 22 and ACC 4, respectively; Braunschweig, Germany). Wien-133 cells (human Burkitt’s lymphoma) were kindly provided by G. Hale (BioAnaLab Limited).

PBMCs derived from patients with CLL were commercially obtained from Discovery Life Sciences. Characteristics of patients with CLL are summarized in Supplementary Table 9. Buffy coats from healthy human donors and complement-competent, pooled NHS (AB positive) were obtained from Sanquin. Whole blood samples from healthy human volunteers were freshly obtained from the University Medical Center Utrecht. Commercially available patient-derived PBMCs and healthy donor blood(-derived) samples were collected at the site of the vendor after patients provided their written and informed consent in accordance with the declaration of Helsinki. All vendors maintained strict ethical compliance, including fully deidentified materials and stringent Institutional Review Board and Ethics Committee compliance. Purified C1q protein and C1 complex were obtained from Quidel and Complement Technology, respectively.

### Whole blood cytotoxicity

Cytotoxicity assays were performed with healthy human donor blood samples that were hirudin anticoagulated, or EDTA anticoagulated and recalcified using 5 mM CaCl_2_ (Sigma Aldrich) for 30 min in the presence of 10 µg ml^−1^ hirudin (Genscript) to preserve or restore complement activity, respectively. While hirudin anticoagulated blood samples were preferred for this study due to optimal preservation of complement activity and enabling ADCC activity, availability of such samples was limited due to discontinuation of the product. Recalcified EDTA anticoagulated blood samples were used as an alternative whole blood source, albeit with limited incubation times (<4 h) to prevent blood clotting. For the hirudin anticoagulated blood samples analyzed in Supplementary Fig. 2 and in Fig. 3, whole blood was incubated with antibodies for 18 h (to enable detection of both CDC and ADCC/ADCP-mediated depletion of cell subsets) at 37 °C/5% CO_2_. After 18 h incubation, red blood cells were lysed first and next samples were stained as described above, before measuring on an LSR Fortessa (BD Biosciences) flow cytometer. For the recalcified, EDTA anticoagulated blood samples used in Fig. 6 and Supplementary Fig. 6, whole blood was incubated with antibodies for 45 min (to enable detection of CDC) at 37 °C/5% CO_2_. After 45 min of incubation, samples were stained for 30 min at 4 °C with fluorochrome-labeled lineage-specific antibodies and fixable viability stain (FVS-BV510; BD Biosciences) to characterize cell subsets and dead or dying cells, respectively. Next, red blood cells were lysed (lysis buffer: 10 mM KHCO_3_, 0.01 mM EDTA and 155 mM NH_4_Cl) and cells were optionally fixed in 2% PFA before measuring on a flow cytometer. Cell markers used to define cell populations were: CD45-PerCP (Biolegend), CD66b-PE-Cy7 (Biolegend), CD3-eFluor450, CD4-APC-eFluor780 (eBioscience) and CD19-BV711 (Biolegend). For Fig. 6 and Supplementary Fig. 6, additional cell markers included were: CD16-BV605 (Biolegend), CD56-PE-CF594 (BD Biosciences) and CD8-AF700 (Biolegend). The gating strategy used to define cell populations is described in Supplementary Fig. 7a. Cytotoxicity was calculated as the fraction (%) of cells remaining after treatment relative to a nontreated control sample (100%).

### C1q binding

Wien-133 cells were opsonized with antibody serial dilutions for 15 min at 37 °C. Subsequently, cells and antibodies were put on ice, purified human complement component C1q (2.5 µg ml^−1^) was added and incubated for 45 min. After washing, C1q binding was detected using a fluorescein isothiocyanate (FITC) conjugated rabbit antihuman C1q antibody (Dako) and quantified as the FITC geometric mean fluorescent intensity (gMFI) determined using an iQue Screener flow cytometer (Sartorius).

### CDC

CDC assays were performed using tumor cells incubated with antibody concentration series or a fixed antibody concentration as indicated, for 45 min at 37 °C in the presence of NHS (20% final concentration) as source of complement. Killing was calculated as the fraction of propidium iodide (PI)-positive cells (%) determined by an iQue Screener flow cytometer for tumor B cell lines, and as the fraction of TO-PRO-3-positive cells (%) determined by an LSR Fortessa flow cytometer for CD19^+^ CLL B cells.

### FcγR binding

Binding of antibody variants to the monomeric extracellular domain (ECD) of FcγRIA (FCGR1AECDHis) and to dimeric ECDs of FcγRIIA allotype 131H (diFCGR2AH-HisBAP), FcγRIIA allotype 131R (diFCGR2AR-HisBAP), FcγRIIIA allotype 158F (diFCGR3AF-HisBAP) and FcγRIIIA allotype 158V (diFCGR3AV-HisBAP) was tested in enzyme-linked immunosorbent assays (ELISAs)^51^. For binding to dimeric FcγR variants, 100 µl of goat antihuman F(ab’)_2_ (1 μg ml^−1^) was added per well for coating overnight at 4 °C. After washing the plates, nonspecific binding was blocked for 1 h at room temperature by adding 200 µl per well PBS/0.2% BSA. With washings in between incubations, plates were sequentially incubated with 100 µl of 20 µg ml^−1^ antibody variants in PBS with Tween with 0.2% BSA buffer for 1 h at room temperature, 100 µl of the recombinant dimeric FcγR constructs (1 µg ml^−1^) for 1 h at room temperature and 100 µl of streptavidin-labeled Poly-HRP (1:10,000) for 30 min at room temperature. Development was performed for 10–30 min with 1 mg ml^−1^ ABTS (Roche). To stop the reactions, 100 µl per well of 2% oxalic acid was added. Absorbance was measured at 405 nm in a microplate reader (BioTek) using BioTek Gen5 V1.04.5 software. To detect binding to monomeric FcγRIa, plates were coated with monomeric His-tagged FCGR1A ECD and after antibody incubation, goat antihuman-kappaLC-HRP (1:5,000) was used as detection antibody.

### FcγR activation

Activation of FcγRIIa- (allotype H-131) and FcγRIIIa-mediated (allotype V-158) intracellular signaling was quantified using Luminescent Reporter Bioassays (Promega), according to the manufacturer’s recommendations.

### ADCC

ADCC was assessed using a DELFIA EuTDA Cytotoxicity assay (PerkinElmer) according to the manufacturer’s recommendations. Briefly, Wien-133 target cells were loaded with BATDA reagent, and 1 × 10^4^ cells were incubated with antibody serial dilutions and human healthy donor PBMCs (isolated from buffy coats through centrifugation using Leucosep tubes according to the manufacturer’s instructions; Greiner Bio-One, catalog no. 227288) as effector cells, at a 100:1 effector to target ratio, for 2 h at 37 °C in a total volume of 160 µl. After incubation and centrifugation, 20 µl of supernatant was transferred to a 96-well plate, 200 µl of Europium Solution was added and the mixture was incubated for 15 min at room temperature while shaking. EuTDA release and time-resolved fluorescence was measured on an EnVision Multilabel Reader (PerkinElmer). Maximal and spontaneous release were determined using target cells incubated with 0.1% Triton X-100 or target cells in medium without effector cells, respectively. Specific release was calculated as:

$${{{\mathrm{\% }}}}\;{\mathrm{specific}}\;{\mathrm{release}} = 100 \times \frac{{\left( {{\mathrm{counts}}\;{\mathrm{release}}\;{\mathrm{sample}} - {\mathrm{counts}}\;{\mathrm{spontaneous}}\;{\mathrm{release}}} \right)}}{{\left( {{\mathrm{counts}}\;{\mathrm{maximal}}\;{\mathrm{release}} - {\mathrm{counts}}\;{\mathrm{spontaneous}}\;{\mathrm{release}}} \right)}}$$

### ADCP

ADCP assays were performed as described in ref. ^52^. In short, human CD14^+^ monocytes were obtained from healthy donor PBMCs (isolated from buffy coats through centrifugation using Leucosep tubes according to the manufacturer’s instructions) through positive isolation using CD14 MicroBeads (Miltenyi Biotec) according to the manufacturer’s instructions. Monocytes were cultured in culture medium (CellGenix GMP DC serum-free medium with 50 ng ml^−1^ M-CSF) in Nunc dishes with UpCell surface (Thermo Fisher Scientific) at 37 °C/5% CO_2_ for 7–8 days to obtain human monocyte-derived macrophages (h-MDM). h-MDMs were characterized by flow cytometry for expression of myeloid- and macrophage-specific maturation markers (Supplementary Table 1). ADCP was determined using Raji cells labeled with calcein AM (Life Technologies) according to the manufacturer’s instructions and opsonized with antibodies for 15 min at 37 °C. h-MDM were added at an effector to target (E:T) ratios of 2:1 and incubated for 4 h at 37 °C/5% CO_2_. After incubation, tumor cells and h-MDM were stained for surface markers using fluorochrome-conjugated antibodies (Supplementary Table 2) for 30 min at 4 °C and analyzed on an LSR Fortessa flow cytometer. The gating strategy used to define cell populations is described in Supplementary Fig. 7b. ADCP was calculated as the fraction of CD11b^+^/calcein AM^+^/CD19^−^ cells within the total h-MDM (CD11b^+^) cell population.

### Target expression

Expression levels of cellular markers were determined using an indirect immunofluorescence assay (QIFIKIT, Agilent Technologies and Human IgG Calibrator kit, BioCytex) according to the manufacturer’s instructions.

### PBMC activation

PBMC CDC assays were performed as follows. Human PBMCs from eight healthy donors (isolated from buffy coats through centrifugation using Leucosep tubes according to the manufacturer’s instructions) were incubated with antibodies in culture medium (Iscove’s Modified Dulbecco’s Medium (IMDM) supplemented with 1 U ml^−1^ penicillin and 1 µg ml^−1^ streptomycin, Lonza) and 20% (final concentration) pooled NHS (Sanquin) as a source of complement, in three individual samples per antibody. After 20–24 h incubation at 37 °C/5% CO_2_, culture supernatant was collected and stored at −20 °C until further analysis. Cytokine quantitation was performed on the culture supernatants using the U-PLEX Proinflam Combo 1 Human kit and Discovery Workbench software v.4.0 (Meso Scale Diagnostics), according to the manufacturer’s instructions. Two donors were excluded based on a high induction of cytokine production by isotype controls.

The gating strategy used to define cell populations was essentially the same as described for whole blood cytotoxicity analysis, and is detailed further in Supplementary Fig. 7. In addition, PBMCs were stained for cell surface markers CD25 (CD25-PE, eBioscience) and CD69 (CD69-FITC, BD Biosciences). Staining was performed for 30 min at 4 °C and analyzed on an LSR Fortessa X20 flow cytometer (BD Biosciences).

### FRET

Proximity-induced FRET was determined by measuring energy transfer between AF555-conjugated donor and AF647-conjugated acceptor antibodies incubated with cells^9,26^. Briefly, purified B cells (isolated from buffy coats using Dynal Dynabeads Untouched Human B cell isolation kit (Life Technologies) according to the manufacturer’s instructions) were incubated with AF555-conjugated donor mAbs and/or AF647-conjugated acceptor mAbs in the presence or absence of purified human C1q (Quidel, 2.5 µg ml^−1^) or C1 (Complement Technology, 2.42 µg ml^−1^). gMFI values were measured using an LSR Fortessa flow cytometer by recording events at 585/42 nm (FL2, donor AF488) and ≥670 nm (FL3), both excited at 488 and at 660/20 nm (FL4, acceptor AF647), excited at 635 nm. Unquenched donor fluorescence intensity was determined with cells incubated with AF555-conjugated donor mAbs, and nonenhanced acceptor intensity was determined with cells incubated with AF647-conjugated acceptor mAbs. Proximity-induced FRET was determined by measuring energy transfer between cells incubated with AF555-conjugated donor and AF647-conjugated acceptor mAbs. gMFI values allowed calculation of FRET according to the following equation:

$${\mathrm{Energy}}\;{\mathrm{transfer}}\;\left( {{\mathrm{ET}}} \right) = {\mathrm{FL}}3\left( {D,\;A} \right) - \frac{{{\mathrm{FL}}2(D,A)}}{{\left( a \right)}} - \frac{{{\mathrm{FL}}4(D,A)}}{{\left( b \right)}}$$

where *a* is FL2(D)/FL3(D), *b* is FL4(A)/FL3(A), *D* is donor, *A* is acceptor and FLn (*D*, *A*) are donor + acceptor. ET values obtained were normalized:

$${\mathrm{Normalized}}\;{\mathrm{ET}}\;({{{\mathrm{\% }}}}) = 100 \times \frac{{{\mathrm{ET}}}}{{{\mathrm{FL}}3(D,A)}}$$

### Viability assay

Cell viability was determined using a CellTiter-Glo luminescent cell viability assay, according to the supplier’s protocol (Promega). Cells were seeded in white OptiPlates (PerkinElmer) and allowed to adhere overnight at 37 °C. The following day, antibody serial dilutions and purified human C1q (Complement Technology, 2.5 µg ml^−1^) were added and incubated for 3 days at 37 °C. Then 5 µM staurosporine (Sigma no. S6942) treated cells and untreated cells were included as positive and negative controls of cell death induction, respectively. After incubation, Luciferin Solution Reagent was added and plates were incubated for 1.5 h at 37 °C. Luminescence was measured on an EnVision Multilabel Reader. The percentage of viable cells was calculated using the following formula:

$${\mathrm{Viable}}\;{\mathrm{cells}}\; (\%) = 100 \times \frac{{T}-{P}}{{V}-{P}}$$

where *T* is luminescence of the test sample, *P* is luminescence of staurosporine control sample and *V* is the luminescence of the medium control sample.

### Human FcRn binding

Kinetic rate and affinity constants were determined using a Biacore 8K SPR system (Cytiva) equipped with a Biacore Series S Sensorchip CM5 (Cytiva catalog no. 29104988) at 25 °C in running buffer of PBS-P+ (10×, Cytiva catalog no. 28995084; diluted and pH adjusted to a final composition of 20 mM phosphate pH 6.0, 137 mM NaCl, 2.7 mM KCl, 0.05% Tween-20). Briefly, anti-His-tag antibody (Cytiva catalog no. 28995056) was covalently linked in all flow cells by NHS/EDC coupling (Cytiva, catalog no. BR100050) according to supplier instructions. After activation, anti-His-tag antibody (50 μg ml^−1^) in 10 mM sodium acetate pH 4.5 (His Capture Kit) was coupled for 420 s at a flow rate of 10 µl min^−1^. After a minimum of 1 h of stabilization in running buffer, 5 nM His-tagged FcRn (Sino Biological, catalog no. CT071-H27H-B) in running buffer was captured for 60 s at 10 µl min^−1^ to capture levels of 45–60 resonance units only in active flow cells, while running buffer was injected in reference flow cells. A twofold, eight-step dilution series of indicated IgG1 molecules (75 nM to 0.29 nM) was prepared in running buffer. IgG dilution series were injected in reverse order in both flow cells for 120 s at 30 μl min^−1^, with a 300 s dissociation phase at 30 μl min^−1^. Samples were injected in a multi-cycle manner over freshly captured FcRn per cycle, by a single regeneration of capture surfaces with 10 mM glycine, pH 1.5 (Cytiva catalog no. BR100354), for 30 s at 30 μl min^−1^. Three independent blank cycles were injected per interaction at the start of the assay, the third of which was used during double referencing. Data were processed and analyzed using Biacore 8K Evaluation Software (v.3.0.12) (GE Healthcare Bio-Sciences Corporation). Responses in the reference cell were subtracted from the active cell to yield reference-subtracted data. A blank sensorgram (recorded in the active cell before injection of IgG) was subtracted from reference-subtracted data to yield double-referenced data. Double-referenced data were fit to a 1:1 binding model to determine the apparent association (*k*_a_) and dissociation (*k*_d_) rate constants. The apparent equilibrium dissociation constant, or affinity constant, *K*_D_, was calculated as *K*_D_ = *k*_d_/*k*_a_.

### Animals

Animal experiments were performed in compliance with the Dutch animal protection law (WoD) translated from the directives (2010/63/EU) and if applicable, the Code of Practice ‘animal experiments for cancer research’ (Inspection V&W, Zutphen, Netherlands, 1999) and were approved by the Dutch Central Committee for animal experiments and by the local ethical committee. Animals were housed and handled in accordance with good animal practice as defined by the Federation of European Laboratory Animal Science Associations, in an association for assessment and accreditation of laboratory animal care and an ISO 9001:2000 accredited animal facility (GDL, Utrecht, Netherlands).

### Pharmacokinetic analysis

Pharmacokinetic studies were performed using 11–12-week-old female tumor-free C.B-17/IcrHan Hsd-Prkdcscid mice (SCID, Envigo). Mice were injected intravenously (IV) with a single dose of 500 µg of each test reagent per mouse (*n* = 3). Blood samples were drawn from the saphenous vein at 10 min, 4 h and 1, 2, 7, 14 and 21 days after antibody administration and collected into heparin-containing vials. Vials were centrifuged (10 min at 14,000*g*) to separate plasma from cells and plasma was stored at −20 °C until further use. Total human IgG concentration in plasma samples was analyzed by ELISA. Plates were coated overnight at 4 °C with 2 μg ml^−1^ of an in-house generated antihuman IgG directed mouse-IgG2a recombinant Fab fragment in PBS, and plasma human IgG was detected by a peroxidase-conjugated AffiniPure goat antihuman IgG Fcγ-specific antibody (Jackson, catalog no. 109-035-098). Absorbance was measured at 405 nm in a microplate reader (BioTek) and processed using BioTek Gen5 V1.04.5 software. Area under the curve (AUC) up to day 21 was determined using GraphPad Prism and clearance was calculated as (Dose (mg kg^−1^) × 1,000/AUC).

### In vivo﻿ proof of concept studies

Human PBMCs were isolated from buffy coats obtained from healthy donors, as described above, and were frozen overnight at −80 °C and stored in liquid nitrogen after 24 h. On thawing, monocytes and Natural Killer cells were depleted using CD14 and CD56 MicroBeads (Miltenyi, catalog no. 130-050-201 9CD14) and 130-050-401 (CD56)) according to the manufacturer’s protocol. One day before the study, NSG mice (NOD.C.B-17-Prkdc scid/J mice; Charles River Laboratories; female; age 7–8 weeks) were injected intraperitoneally with 8 mg IVIg (Octagam, Pharmacy of Veterinary Medicine, Utrecht University, catalog no. 5430000803496). NSG mice were injected intravenously with antibody mixtures (combined dose of both antibody components was 1.0 mg kg^−1^) and immediately thereafter 8 × 10^6^ human T and B cells from three separate donors were injected intraperitoneally. The next day, mice were euthanized and the human cells were recovered by peritoneal lavage. The number of human cells was analyzed by hCD45(^+^) staining, T cells were identified as hCD3(^+^)/hCD2(^+^), B cells as hCD19(+)/hCD22(+), and the number of cells recovered was expressed relative to a fixed number of beads added at the start of the staining. Deposited C3 was detected by a directly labeled mAb against mouse C3 (details in Supplementary Table 5). Significance of comparisons between log-transformed cell counts or mean fluorescence intensities of C3 deposition were assessed by one-way analysis of variance after Welch correction for nonequal s.d., followed by Dunnett’s T3 multiple comparisons test.

Human pluripotent stem cell (HPSC) -humanized NSG (NSG-HIS) mice were purchased from Jackson (female, age 16–18 weeks after quality control of immune reconstitution) from three HPSC donors. Humanization levels were confirmed on arrival by flow cytometry (Supplementary Table 8), and randomization was performed based on human CD45(^+^) counts and the donors. One day before the study all animals were injected intraperitoneally with 8 mg IVIg (Octagam). NSG-HIS mice were treated intravenously with antibody mixtures at a combined dose of 0.5 mg kg^−1^. Mice were euthanized the next day. Blood samples were incubated with fluorescently labeled antibodies (Supplementary Table 8) and red blood cell Lysis buffer (BioLegend, catalog no. 420302). Peritoneal cells were also incubated with fluorescently labeled antibodies after washing in PBS. CountBright Beads (Invitrogen, catalog no. C36950) were added to blood and peritoneal cell samples to determine the number of human cells and C3 fixation by flow cytometry on a FACS Fortessa (BD). Significance of comparisons between log-transformed cell counts or mean fluorescence intensities of C3 deposition were assessed by one-way analysis of variance after Welch correction for nonequal s.d.s, followed by Dunnett’s T3 multiple comparisons test.

All fluorescently labeled antibodies specific for the cell markers used to define cell populations are listed in Supplementary Table 8. The gating strategy used to define cell populations is described in Supplementary Fig. 7.

### Super-resolution microscopy sample preparation

Wien-133 suspension cells were cultured in IMDM supplemented with 10% heat-inactivated fetal bovine serum, 5 U ml^−1^ penicillin, 0.05 mg ml^−1^ streptomycin and 2 mM l-glutamine at 37 °C and 5% carbon dioxide. Washes described below were performed by pelleting cells at 0.1 RCF using an Eppendorf centrifuge (model no. 5415R) followed by buffer exchange. Cells were washed once with warm IMDM and aliquoted into 1.5 ml Eppendorf tubes at 4 × 10^6^ cells per treatment group. Cells were incubated with 5 µg ml^−1^ of total IgG for each antibody combination plus 5 µg ml^−1^ C1q in IMDM for 15 min at 37 °C. After antibody opsonization, cells were washed once with PBS and fixed with 4% paraformaldehyde (PFA) plus 0.2% glutaraldehyde in PBS at room temperature for 2 h. Fixation was halted by removing the PFA/GA fixative via centrifugation, followed by two washes with 10 mM Tris and then blocked for 15 min with 5% BSA/PBS. CD52- and CD20-directed antibody variants used for super-resolution imaging contained an HA-tag at the C terminus of the light chain, allowing for detection using AF647-labeled anti-HA antibody (Novus Biological NB600-363AF647). Cells were incubated with anti-HA-AF647 at 8 µg ml^−1^ in 2% BSA/PBS for 1 h at room temperature. Labeled cells were washed with PBS and postfixed with 4% PFA for 10 min at room temperature, washed twice with 10 mM Tris and suspended in PBS until imaging.

### Super-resolution microscopy image acquisition

For dSTORM imaging, 25 mm glass coverslips (no. 1.5, Warner Instruments, no. CS-25R15) were piranha etched (96% H_2_SO_4_ + 30% H_2_O_2_) and treated with 0.1% NABH_4_ for 5 min to quench background fluorescence. Coverslips were dried and glow discharged before incubation with 0.01% poly-l-lysine solution plus 0.01% poly-d-lysine hydrobromide (Sigma nos. P4707, P1149) for 3 h at room temperature. Coverslips were rinsed with water and dried before storage at 4 °C. Poly-l/d-lysine coated coverslips were secured into custom-made imaging chambers and rehydrated with PBS for 10 min at room temperature. After removal of PBS used for coverslip hydration, 4 × 10^6^ Wien-133 cells in 1 ml of PBS were allowed to settle onto the coverslip for 8 h at room temperature. Cells were gently washed twice with PBS before addition of 1.5 ml of fresh dSTORM imaging buffer (50 mM Tris, 10 mM NaCl, 10% w/v glucose, 168.8 U ml^−1^ glucose oxidase (Sigma no. G2133), 1,404 U ml^−1^ catalase (Sigma no. C9322) and 60 mM 2-aminoethanethiol (MEA), pH 8.0). The chamber was sealed by securing a clean 25 mm coverslip over the top.

dSTORM imaging was performed using a custom-built microscope equipped with a 1.35 NA silicon oil immersion lens (UPLSAPO100XS, Olympus) and an sCMOS camera (C11440-22CU, Hamamatsu). Excitation was with a 647 nm fiber laser (2RU-VFL-P-500-647-B1R, MPB Communications). Emission light was collected with a 708/75 nm band pass filter (FF01-708/75-25, Semrock). Brightfield registration^53^ was performed before each sequence using a 660 nm LED (M660L3, Thorlabs) illumination lamp and a 3D piezo sample stage (MAX341/M, Thorlabs). A total of 100,000 frames were collected for each cell (20 sequences of 5,000 frames each) at 20 frames per second and approximately 50 mW of excitation laser power.

### Super-resolution microscopy image reconstruction and data analysis

dSTORM images were analyzed and reconstructed with custom-built functions in MATLAB R2019b software (MathLinks). For each image frame, subregions were selected based on local maximum intensity. Each subregion was then fitted to a finite pixel Gaussian intensity distribution using a maximum likelihood estimator^54^. Fitted results were accepted or rejected based on log-likelihood ratio, the fit precision that was estimated using the Cramér–Rao lower bound values for each parameter, as well as intensity and background cutoffs^55^ between localizations in close spatial-temporal proximity that were possibly from the same blinking event were connected if the null hypothesis that they originated from a fluorophore at the same location was not rejected at a level of significance of 0.01.

Localizations were used to make estimates of the underlying number and locations of the fluorescence emitters using the Bayesian grouping of localizations (BaGoL) algorithm^56^. We carried out BaGoL analysis using the MATLAB implementation of BaGoL included with smite (https://github.com/LidkeLab/smite/) v.0.1.0 at the UNM Center for High-Performance Computing using MATLAB R2019b. The locations and uncertainty given by the maximum a posteriori number of emitters output of the BaGoL algorithm were used for further analysis (Supplementary Fig. 4). The well-separated fluorophores in the sparsely labeled IgG1-b12-RGE-HA samples were used to estimate the blinking statistics for the anti-HA-AF647 probe. From the blinking statistics of individual antibodies, we found an optimal precision inflation (SE_adjust) parameter of 9 nm. Blinking statistics were modeled with an exponential distribution with mean value of 5.131 and 4.333 blinking events per antibody probe for the two lots of anti-HA-AF647.

ROI of size 2 × 2 μm were selected from the set of images, from which statistics for the number of localizations and nearest neighbor distances were collected per ROI. The nearest neighbor distance in each ROI was computed with locally written MATLAB R2019Bb software using ‘knnsearch’, producing the distance from each localization to its nearest neighbor, and so inferring an average localization density.

### Nonmicroscopy data processing

LSR Fortesssa flow cytometry data were processed using FACSDiva v.8 and v.9.0 software (BD). Sartorius iQue flow cytometry data were processed using iQue ForeCyt v.6.2 and v.8.1 software (Sartorius). Flow cytometry data were analyzed using FlowJo V10 software (BD). Graphs were plotted and analyzed using GraphPad Prism v.8.0 (DotMatics). EnVision data were processed using EnVision Workstation 1.13.3009.1401 software (Perkin Elmer). Graphs were plotted and analyzed using GraphPad Prism v.8.0. Dose-response curves were generated using best-fit values of nonlinear dose-response fits using log-transformed concentrations. All data shown are representative of at least three independent replicate experiments or three individual human donors tested. The mean AUC ± standard deviation (s.d.) was determined for all available dose-response analyses and is summarized in Supplementary Table 3. Dose-response data from multiple experimental repeats were pooled, concentrations were log-transformed and the resulting AUC values were normalized relative to the positive control indicated (100%) and negative control nonbinding antibody IgG1-b12 (0%).

### Reporting summary﻿

Further information on research design is available in the Nature Research Reporting Summary linked to this article.

## Online content

Any methods, additional references, Nature Research reporting summaries, source data, extended data, supplementary information, acknowledgements, peer review information; details of author contributions and competing interests; and statements of data and code availability are available at 10.1038/s41587-022-01384-1.

## Supplementary information

Supplementary Information Supplementary Figs. 1–7 and Tables 1–9.

Reporting Summary.

## Data Availability

Sequences of the variable domains and constant domains of recombinant antibodies tested herein were described in patent WO 2019/211472. The datasets generated during and/or analyzed during the current study are available from the corresponding author upon reasonable request.

## Code availability

The algorithms BaGoL and knnsearch are available from M.J.W. and K. Lidke via wester@unm.edu.
