# Supplementary material for: Logic-gated antibody pairs that selectively act on cells co-expressing two antigens
Source: Nat Biotechnol. 2022 Jul 25;40(10):1509–19. doi: 10.1038/s41587-022-01384-1 (PMC9546771; doi:10.1038/s41587-022-01384-1)
Supplement: Supplementary file 1 — Supplementary Figs. 1–7 and Tables 1–9. [file 41587_2022_1384_MOESM1_ESM.pdf]

nature portfolio

https://doi.org/10.1038/s41587-022-01384-1

Supplementary information

Logic-gated antibody pairs that selectively act on cells co-expressing two antigens

In the format provided by the authors and unedited

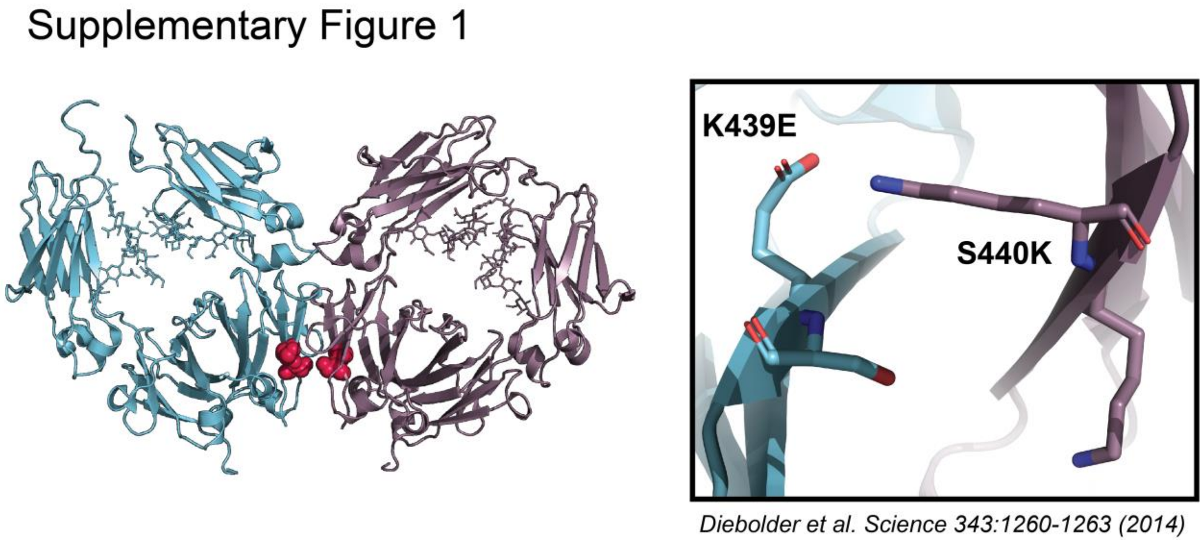

Supplementary Figure 1. Fc-Fc interaction interface model. Left: Ribbon diagram of two Fc segments with residues (Lys^{439} and Ser^{440}) critical for Fc-Fc interactions indicated in pink. Right: modeled interactions of a K439E (Lys^{439} → Glu) mutant facing the S440K (Ser^{440} → Lys) mutant on the complementary Fc segment of a neighboring antibody. Figure adapted from Diebolder et al. Science 343:1260-1263 (2014).

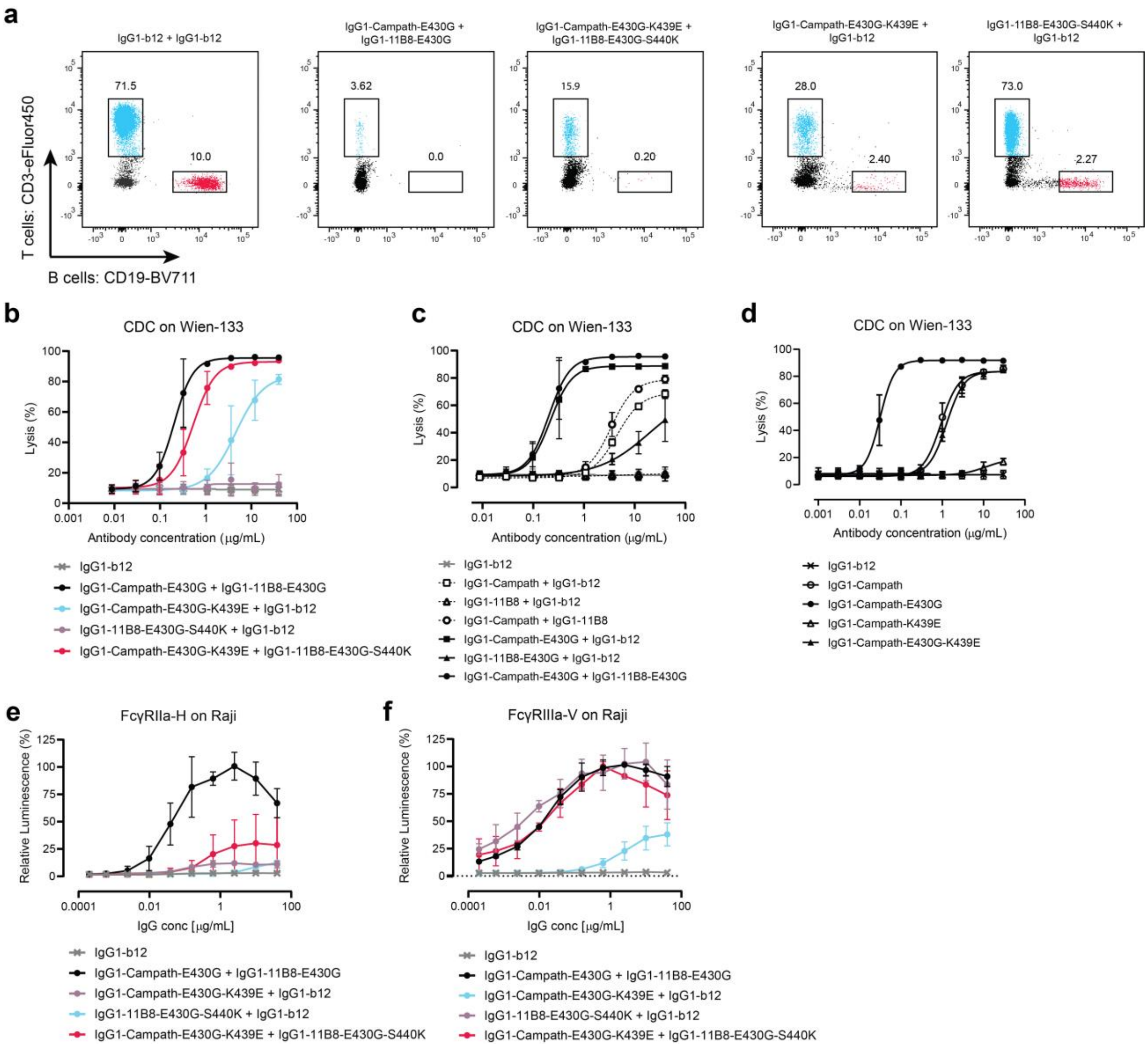

Supplementary Figure 2. Broad depletion of hematological cell subsets by CD52 and CD20 antibody combinations. (a) B- and T-cell cytotoxicity was assessed in healthy human whole blood incubated with non-binding control antibody IgG1-b12 or equimolar mixtures (final concentration 10 µg/mL total IgG) of IgG1-Campath and IgG1-11B8 antibody variants for 18 hours at 37 °C and analyzed by flow cytometry. The fraction (%) of B- or T cells remaining within the lymphocyte population (CD66b-) from one representative donor (out of 5 tested donors) is shown. (b-c) CDC of Wien-133 cells in dose-response titrations of IgG1-Campath and IgG1-11B8 antibody variants and combinations. Error bars indicate mean and standard deviation (SD) over n=3 independent experiments. (d) Residual CDC by Fc-Fc interface mutant CD52 antibody variants. CDC was assessed on Wien-133 cells opsonized with a concentration series of different

IgG1-Campath antibody variants. Data was averaged over three experiments, normalized to IgG1-b12 (0 % lysis) and IgG1-Campath-E430G (100% lysis), and is presented as the area under the curve (AUC). Error bars indicate average and standard deviation over n=3 experiments. (e-f) Dose-dependent activation of FcγRIIa- (e) and FcγRIIIa-mediated (f) intracellular signaling by IgG1-Campath and IgG1-11B8 antibody variants was quantified by a luminescent reporter bioassay using Raji target cells and Jurkat T-effector cells expressing FcγRIIa H131 (e) or FcγRIIIa V158 (f). Luminescence values were normalized to the value for 10 μg/mL IgG1-11B8-E430G + IgG1-b12 prior to pooling, and are presented as the percentage relative luminescence. Error bars indicate mean and standard deviation over n=3 independent experiments. (b-f) See Supplementary Table 3 for statistical analysis of AUC values.

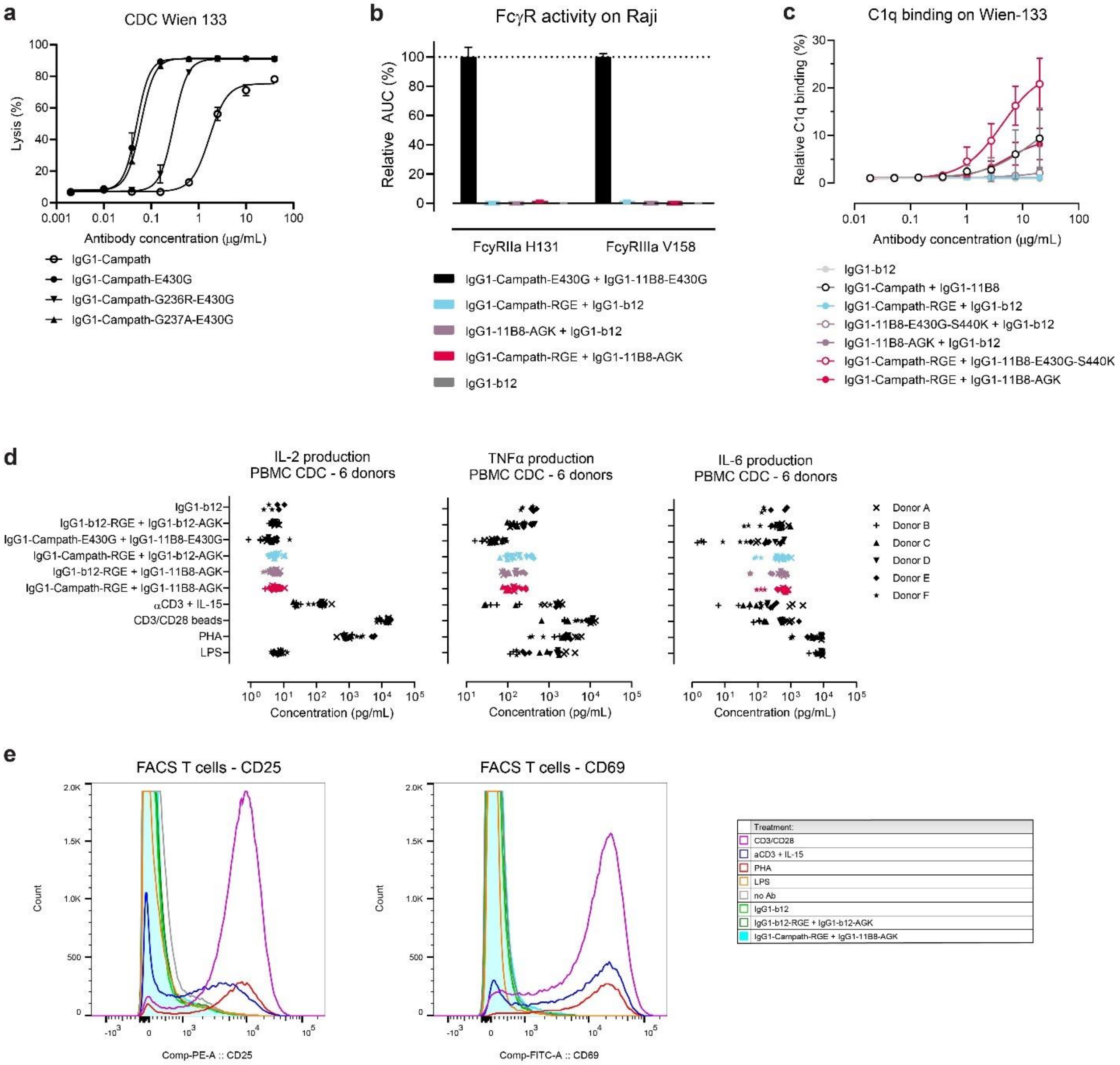

Supplementary Figure 3. Functional characterization of C1q- and FcγR-binding modulated IgG1 antibody variants. (a) CDC was assessed using Wien-133 cells opsonized with a concentration series of different IgG1-Campath antibody variants. Data was averaged over three experiments, normalized to IgG1-b12 (0 % lysis) and IgG1-Campath-E430G (100% lysis), and is presented as the area under the curve (AUC). (b) Activation of FcγR-mediated signaling was assessed in a Luminescent Reporter Bioassay using Raji target cells and Jurkat T-effector cells. Luminescence values were normalized to the value of 10 µg/mL IgG1-11B8-E430G + IgG1-b12 prior to pooling. Area under the curve (AUC) values were averaged over three experiments,

normalized to IgG1-b12 (0 % activation) and a mixture of IgG1-11B8-E430G and IgG1-Campath-E430G (100% activation). (c) C1q binding to Wien-133 cells opsonized with a concentration series of IgG1-Campath and IgG1-11B8 mutant antibody variants. Data were normalized to IgG1-b12 (0 % C1q binding) and a mixture of IgG1-11B8-E430G and IgG1-Campath-E430G (100% C1q binding). (a-c) Mean and standard deviation over n=3 independent experiments are shown. See Supplementary Table 3 for statistical analysis of AUC values. (d) (e) PBMCs isolated from the blood of six healthy donors were incubated for 20-24 hours in the presence of 10 µg/mL of each antibody mixture and 20% (V/V) complement-competent normal human serum. A mixture of anti-CD3 and IL-15, anti-CD3 and anti-CD28 antibodies immobilized on beads, PHA, and LPS served as positive control stimuli of different cytokine responses and activation markers. (d) Supernatant cytokine levels were analyzed by MSD QuickPlex Cytokine analysis. IL-2, TNF-α and IL-6 levels were measured for n=6 donors, in three individually incubated samples per donor, collected in two experiments. (e) Flow cytometry analysis of mean fluorescence intensity of T cells from a representative donor, defined as CD45(+)CD66b(-)CD19(-)CD56(-) lymphocytes to circumvent CD3 detection positive control stimuli competing for CD3 binding, stained for CD25 and CD69 expression.

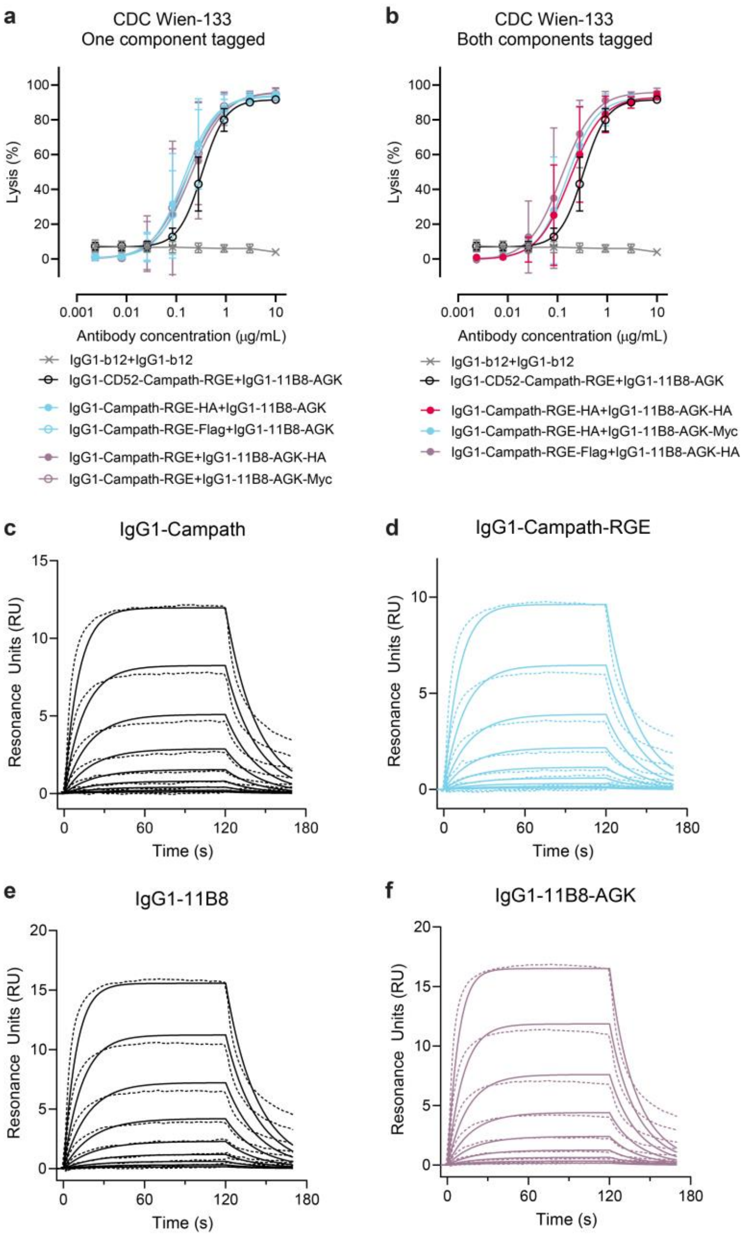

Supplementary Figure 4. Functional and SPR characterization of IgG1 antibody variants. (a-b) CDC was assessed using Wien-133 cells opsonized with a concentration series of IgG1-Campath and IgG1-11B8 antibody variants with different peptide affinity tags recombinantly introduced at the C-terminus of the light chain. Tagged antibody combinations were compared to untagged IgG1-Campath-RGE+IgG1-11B8-AGK. (a) CDC induced by antibody combinations containing a single tagged component. (b). CDC induced by antibody combinations composed of two tagged components. (a-b) Error bars indicate mean and standard deviation over n=3 independent experiments. (c-f) Example SPR sensorgrams of a dose range of IgG1 antibody variants binding to immobilized human FcRn at pH 6.0. Dashed lines indicate double referenced data, solid lines indicate 1:1 binding models fitted to that data. Binding constants were measured in n=3 independent experiments. See Supplementary Table 5 for statistical comparison.

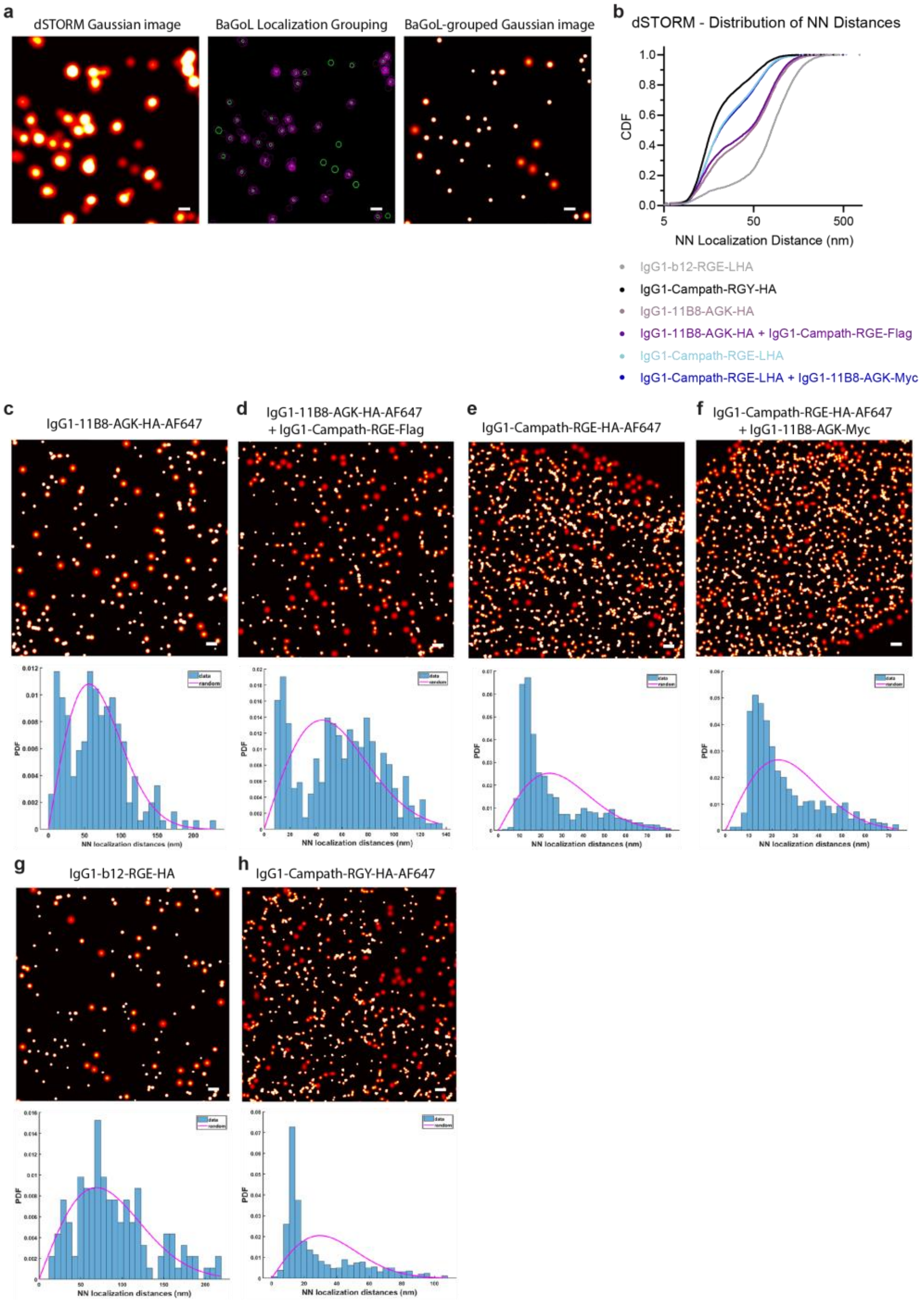

Supplementary Figure 5. Super-resolution microscopy analysis of CD20 and CD52 antibody distributions. (a) Example of analysis workflow, moving from dSTORM Gaussian image (left), to the overlay image (middle) showing dSTORM localizations (magenta) and BaGoL emitter locations (green) and the post-BaGoL Gaussian image (MAPN, right). Scale bars, 200 nm. HA-tagged antibody variants were detected using anti-HA-A647. (b) Nearest Neighbor (NN) distance distribution plotted as a Cumulative Distribution Function (CDF). Underlying data was used to calculate averaged NN distance per ROI shown in Fig. 4d, but here the distribution of all individual NN distances measured is plotted. Consistent with Fig. 4, separation is largest for IgG1-b12-RGE-

HA and smallest for IgG1-Campath variants. Addition of a complementary component did not affect the IgG1-CD52-RGE-HA or IgG1-11B8-AGK-HA NN distance distributions. (c-h) Representative BaGoL-grouped Gaussian image (MAPN) and corresponding NN distance histograms of anti-HA-A647 bound to IgG1-11B8-AGK-HA (c), IgG1-11B8-AGK-HA + IgG1-Campath-RGE-Flag (d), IgG1-Campath-RGE-HA (e), and IgG1-Campath-RGE-HA + IgG1-11B8-AGK-myc (f), IgG1-b12-RGE-HA (g), and IgG1-Campath-RGY-HA (h). Scale bars, 100 nm. The NN distance distribution of IgG1-b12-RGE-HA is consistent with a random distribution (magenta line). Positive control IgG1-Campath-RGY-HA is shifted to smaller separations, indicating clustering.

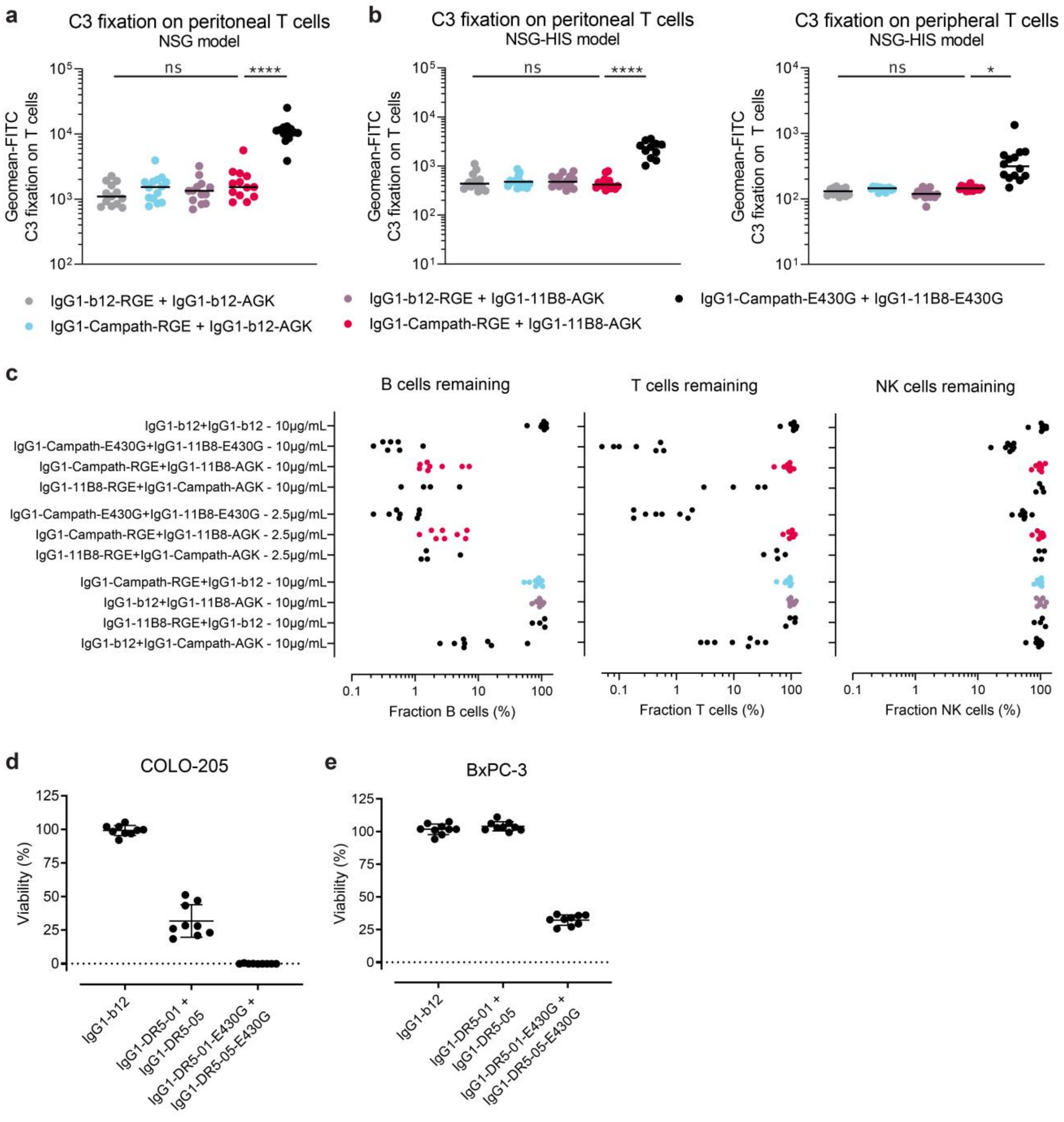

Supplementary Figure 6. Functional activity of self-interaction inhibited and hexamerization-enhanced antibody variants. (a,b) Flow cytometry analyses of mean fluorescence intensity of C3 fragment deposition on human CD3+/CD2+ T cells detected by anti-C3-FITC conjugate. Black lines indicate median values; statistical differences between groups are indicated by *P<0.05, ** P<0.01, *** P<0.001 and **** P<0.0001; n=13 to 14 mice per group (a), resp. 11 to 15 mice per group (b-c); see Supplementary Table 7 for detailed statistical comparisons. Human T cells were recovered by peritoneal lavage from NSG (a), and by peritoneal

lavage and from blood for NSG-HIS mice (b), after 19 hours treatment with the indicated, intravenously injected antibody combinations. (c) Cytotoxicity induced by IgG1-Campath and IgG1-11B8 antibody mixtures (2.5 or 10 µg/mL final concentration) incubated for 45 minutes in healthy human whole blood, as analyzed by flow cytometry. The fraction (%) of CD19(+) B cells, CD3(+) T cells, and CD56(+)CD3(-) NK cells remaining within the CD45(+)CD66b(-) lymphocyte population is shown for three individual donors relative to a non-treated (no antibody) control sample. (d) COLO-205 and (e) BxPC-3 cells were incubated with wild-type and hexamerization-enhanced (E430G) anti-DR5 IgG1-DR5-01 and IgG1-DR5-05 antibody variants (final concentration 20 µg/mL) in the presence of 2.5 µg/mL purifed human C1q and cell viability (%) was measured after 72 hours. Error bars indicate mean and standard deviation over n=9 measurements collected in 3 independent experiments. See Supplementary Table 3 for statistical analysis.

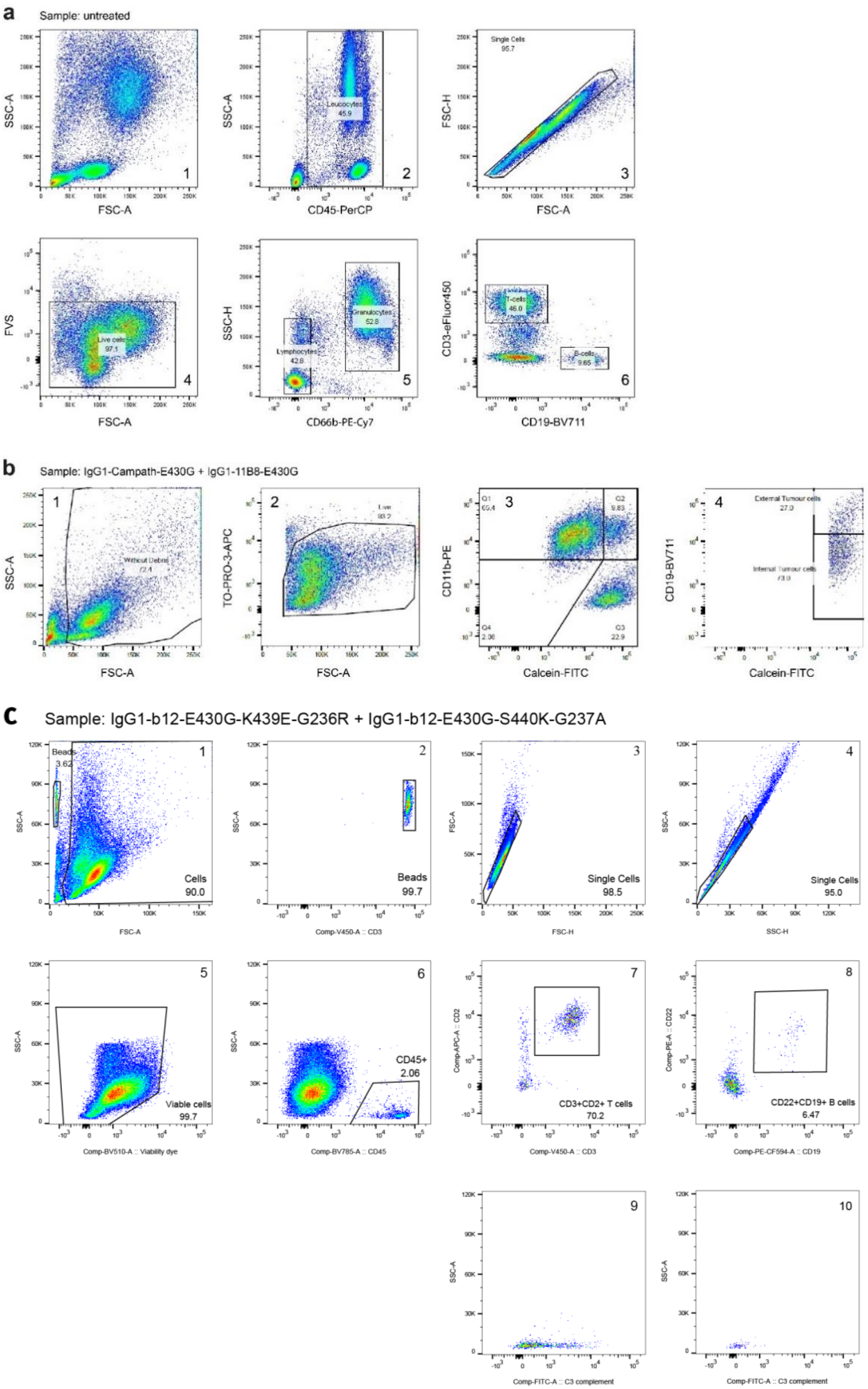
a
Sample: untreated
SSC-A
FSC-A
CD45-PerCP
Leucocytes
FSC-H
Single Cells
FVS
Live cells
SSC-H
CD66b-PE-Cy7
Lymphocytes
Granulocytes
CD3-eFluor450
CD19-BV711
T-cells
B-cells
b
Sample: IgG1-Campath-E430G + IgG1-11B8-E430G
Without Debris
TO-PRO-3-APC
Live
CD11b-PE
Calcein-FITC
External Tumour cells
Internal Tumour cells
c
Sample: IgG1-b12-E430G-K439E-G236R + IgG1-b12-E430G-S440K-G237A
Beads
Cells
90.0
Beads
99.7
Single Cells
98.5
Single Cells
95.0
Viable cells
99.7
CD45+
2.06
CD3+CD2+ T cells
70.2
CD22+CD19+ B cells
6.47

Supplementary Figure 7. Flow cytometry gating strategy used to define cell populations by flow cytometry. (a) Example of whole blood cytotoxicity flow cytometry gating strategy, as illustrated for a negative control sample (untreated cells) derived from a healthy human donor. (1) Cells were identified based on forward scatter (FSC) vs. side scatter (SSC). (2) Leukocytes were selected by $CD45^+$ staining. (3) Doublets were excluded by FSC-A vs. FSC-H. (4) Dead cells were excluded using fixable viability stain (FVS). (5) Lymphocytes and granulocytes were separated based on CD66b staining. (6) B- and T cells within the lymphocyte ($CD66b^-$) cell population were identified as $CD19^+$ and $CD3^+$/($CD4^+$, data not shown), respectively. Cytotoxicity was calculated as the fraction (%) of cells remaining after treatment relative to a non-treated control sample (100%).

(b) Example of ADCP flow cytometry gating strategy, as illustrated for a positive control sample (IgG1-Campath-E430G + IgG1-11B8-E430G) with Raji target cells and human monocyte-derived macrophage (h-MDM) effector cells. (1) Cells were identified based on forward scatter (FSC) vs. side scatter (SSC). (2) Dead cells were excluded by fixable viability stain (FVS). (3) Raji target cells and h-MDM effector cells were separated by calcein AM and CD11b staining respectively. (4) CD19 was used as a marker to exclude external (non-phagocytosed) Raji target cells. $CD11b^+$/calcein AM^+/$CD19^-$ cells were identified as h-MDM that phagocytosed Daudi cells. ADCP was calculated as the fraction of $CD11b^+$/calcein AM^+/$CD19^-$ cells within the total h-MDM ($CD11b^+$) cell population.

(c) Example of flow cytometry gating strategy for lavage sample of NSG-HIS model to calculate T- and B-cell depletion and C3 complement fixation, as illustrated for a negative control sample (HxEL1-b12 + HxEL2-b12 treatment). (1) Cells and Counting beads were identified based on forward scatter (FSC) vs. side scatter (SSC). (2) Based on autofluorescence, counting beads were counted to correct accuracy of the flow cytometry. Doublets were excluded by FSC-A vs. FSC-H (3) and SSC-A vs. SSC-H (4). (5) Dead cells were excluded by Fixable Viability Stain FVS510 staining. (6) Human immune cells were separated based on CD45 staining. (7) T-cell population was identified as CD3+ and CD2+ double positive cells. (8) B-cell population was identified as CD19+ and CD22+ double positive cells. C3 complement fixation was detected on the remaining T cells (9) and B cells (10).

Supplementary Table 1. Antibodies used for h-MDM characterization.

**Target**	**Label**	**Target expression**	**Company**	**Clone**	**Cat. No.**
**CD14**	PE-Cy7	Maturation and lineage marker for monocytes/macrophages	BD Pharmingen	M5E2	557742
**CD11b**	PE	General myeloid cell lineage and maturation marker	BD Pharmingen	ICRF44	555388
**CD64**	FITC	Fc□RI (IgG1), expressed on mature antigen-presenting cells including macrophages	Biolegend	10.1	305006
**CD80**	APC	B7-1, expressed on activated antigen-presenting cells, including macrophages	Miltenyi	2D10	130-097-204
**CD163**	BV421	Macrophage sub lineage/maturity marker	Biolegend	GHI/61	333612
**CD206**	BV711	Mannose receptor, macrophage maturity/sub lineage marker	Biolegend	15-2	321136
**FVS**	eFluor660	Staining of dead cells	BD Biosciences		565694

Supplementary Table 2. Antibodies used for identification of cell subsets in ADCP assays.

Target	Label	Target expression	Company	Clone	Cat. No.
**CD11b**	PE	h-MDM	BD Pharmingen	ICRF44	555388
**CD19**	BV711	Tumor B cells (Daudi)	Biolegend	SJ25C1	363026
**TO-PRO-3**	APC	Staining of dead cells	Molecular Probes		T3605

Supplementary Table 3. Area under the curve statistics as determined for multiple dose-response analyses.

Fig.	Sub	Item[*1]	Antibody component 1	Antibody component 2	Assay	Cells	N[*2]	Mean rel. AUC (%)*[3]	SD	95% CI of diff. to PC[*4]	Adjusted P-value*[5]	95% CI of diff. to NC[*6]	Adjusted P-value*[7]
2	b	PC	IgG1-Campath-E430G	IgG1-11B8-E430G	ADCC	Wien-133	6	100.0	18.03			-117.9 to -82.06	<0.0001
2	b	TI	IgG1-Campath-RGE	IgG1-b12	ADCC	Wien-133	6	-2.90	5.40	84.95 to 120.8	<0.0001	-15.05 to 20.84	0.9910
2	b	TI	IgG1-11B8-AGK	IgG1-b12	ADCC	Wien-133	6	-3.44	5.31	85.50 to 121.4	<0.0001	-14.50 to 21.39	0.9805
2	b	TI	IgG1-Campath-RGE	IgG1-11B8-AGK	ADCC	Wien-133	6	1.72	5.21	80.33 to 116.2	<0.0001	-19.67 to 16.22	0.9987
2	b	NC	IgG1-b12	IgG1-b12	ADCC	Wien-133	6	0.00	5.37	82.06 to 117.9	<0.0001		
2	c	PC	IgG1-Campath-E430G	IgG1-11B8-E430G	ADCP	Raji	6	100.0	5.59			-106.5 to -93.54	<0.0001
2	c	TI	IgG1-Campath-RGE	IgG1-b12	ADCP	Raji	6	0.17	1.51	93.37 to 106.3	<0.0001	-6.628 to 6.291	>0.9999
2	c	TI	IgG1-11B8-AGK	IgG1-b12	ADCP	Raji	6	25.79	4.84	67.75 to 80.67	<0.0001	-32.25 to -19.33	<0.0001
2	c	TI	IgG1-Campath-RGE	IgG1-11B8-AGK	ADCP	Raji	6	39.16	5.83	54.38 to 67.30	<0.0001	-45.62 to -32.70	<0.0001
2	c	NC	IgG1-b12	IgG1-b12	ADCP	Raji	6	0.00	1.05	93.54 to 106.5	<0.0001		
2	d	PC	IgG1-Campath	IgG1-11B8	CDC	Wien-133	3	100.0	5.76			-113.7 to -86.29	<0.0001
2	d	TI	IgG1-Campath-E430G	IgG1-11B8-E430G	CDC	Wien-133	3	276.92	12.83	-192.4 to -161.5	<0.0001	-290.6 to -263.2	<0.0001
2	d	TI	IgG1-Campath-RGE	IgG1-b12	CDC	Wien-133	3	-12.51	2.78	97.04 to 128.0	<0.0001	-1.207 to 26.22	0.0808
2	d	TI	IgG1-11B8-AGK	IgG1-b12	CDC	Wien-133	3	3.86	7.82	80.68 to 111.6	<0.0001	-17.57 to 9.857	0.9091
2	d	TI	IgG1-Campath-RGE	IgG1-11B8-AGK	CDC	Wien-133	3	146.83	3.01	-62.29 to -31.36	<0.0001	-160.5 to -133.1	<0.0001
2	d	NC	IgG1-b12	IgG1-b12	CDC	Wien-133	6	0.00	5.17	86.61 to 113.4	<0.0001		
6	b	PC	Tit IgG1-Campath-RGE	Tit IgG1-11B8-AGK	CDC	Wien-133	3	100.0	2.91			ND	ND

6	b	TI	Tit IgG1-Campath-RGE	20 µg/mL IgG1-11B8-AGK	CDC	Wien-133	3	141.7	4.24	-50.01 to -33.39	<0.0001	ND	ND
6	b	TI	20 µg/mL IgG1-Campath-RGE	Tit IgG1-11B8-AGK	CDC	Wien-133	3	122.00	3.38	-30.31 to -13.70	0.0005	ND	ND
6[*9]	d	PC	IgG1-37.3-E430G	IgG1-11B8-E430G	CDC	Raji	9	85.3	4.55			-85.44 to -73.31	<0.0001
6	d	TI	IgG1-37.3-RGE	IgG1-b12	CDC	Raji	3	11.3	5.81	65.38 to 82.54	<0.0001	-14.00 to 3.158	0.3241
6	d	TI	IgG1-11B8-AGK	IgG1-b12	CDC	Raji	3	11.4	6.62	65.24 to 82.40	<0.0001	-14.14 to 3.024	0.3034
6	d	TI	IgG1-37.3-RGE	IgG1-11B8-AGK	CDC	Raji	3	84.3	8.20	-7.591 to 9.569	0.9955	-86.97 to -69.81	<0.0001
6	d	NC	IgG1-b12	IgG1-b12	CDC	Raji	9	5.88	2.60	73.31 to 85.44	<0.0001		
6[*9]	e	PC	IgG1-DR5-01-E430G	IgG1-DR5-05-E430G	Apoptosis	COLO-205	3	-0.353	0.135			94.50 to 107.3	<0.0001
6	e	TI	IgG1-DR5-01-RGE	IgG1-b12	Apoptosis	COLO-205	3	107	4.44	-115.1 to -99.42	<0.0001	-12.77 to 0.05703	0.0526
6	e	TI	IgG1-DR5-05-AGK	IgG1-b12	Apoptosis	COLO-205	3	110	2.42	-117.7 to -102.0	<0.0001	-15.38 to -2.552	0.0046
6	e	TI	IgG1-DR5-01-RGE	IgG1-DR5-05-AGK	Apoptosis	COLO-205	3	1.99	3.79	-10.19 to 5.515	0.8283	92.17 to 105.0	<0.0001
6	e	NC	IgG1-b12	IgG1-b12	Apoptosis	COLO-205	12	101	3.96	-107.1 to -94.71	<0.0001		
6[*9]	e	PC	IgG1-DR5-01-E430G	IgG1-DR5-05-E430G	Apoptosis	BxPC-3	3	44.8	14.4			43.64 to 74.35	<0.0001
6	e	TI	IgG1-DR5-01-RGE	IgG1-b12	Apoptosis	BxPC-3	3	105	2.70	-78.96 to -41.34	<0.0001	-16.51 to 14.20	0.9990
6	e	TI	IgG1-DR5-05-AGK	IgG1-b12	Apoptosis	BxPC-3	3	104	4.75	-77.92 to -40.30	<0.0001	-15.47 to 15.24	>0.9999
6	e	TI	IgG1-DR5-01-RGE	IgG1-DR5-05-AGK	Apoptosis	BxPC-3	3	49.2	17.2	-23.17 to 14.45	0.9165	39.28 to 69.99	<0.0001
6	e	NC	IgG1-b12	IgG1-b12	Apoptosis	BxPC-3	12	104	5.88	-73.87 to -44.13	<0.0001		
S2	b	PC	IgG1-Campath-E430G	IgG1-11B8-E430G	CDC	Wien-133	3	100.0	4.63			-107.0 to -92.98	<0.0001
S2	b	TI	IgG1-Campath-E430G-K439E	IgG1-b12	CDC	Wien-133	3	34.85	5.13	57.22 to 73.09	<0.0001	-41.87 to -27.82	<0.0001

S2	b	TI	IgG1-11B8-E430G-S440K	IgG1-b12	CDC	Wien-133	3	2.54	2.75	89.53 to 105.4	<0.0001	-9.566 to 4.483	0.7404
S2	b	TI	IgG1-Campath-E430G-K439E	IgG1-11B8-E430G-S440K	CDC	Wien-133	3	79.85	4.05	12.22 to 28.08	<0.0001	-86.88 to -72.83	<0.0001
S2	b	NC	IgG1-b12	IgG1-b12	CDC	Wien-133	6	0.00	1.87	93.13 to 106.9	<0.0001		
S2	c	PC	IgG1-Campath-E430G	IgG1-11B8-E430G	CDC	Wien-133	3	100.0	4.63			-105.5 to -94.52	<0.0001
S2	c	TI	IgG1-Campath-E430G	IgG1-b12	CDC	Wien-133	3	90.58	5.76	2.705 to 16.13	0.0039	-97.30 to -83.87	<0.0001
S2	c	TI	IgG1-11B8-E430G	IgG1-b12	CDC	Wien-133	3	15.03	2.90	78.26 to 91.68	<0.0001	-21.74 to -8.319	<0.0001
S2	c	TI	IgG1-Campath	IgG1-b12	CDC	Wien-133	3	25.79	0.81	67.50 to 80.92	<0.0001	-32.50 to -19.08	<0.0001
S2	c	TI	IgG1-11B8	IgG1-b12	CDC	Wien-133	3	-1.41	1.44	94.70 to 108.1	<0.0001	-5.299 to 8.124	0.9839
S2	c	TI	IgG1-Campath	IgG1-11B8	CDC	Wien-133	3	36.11	2.08	57.18 to 70.60	<0.0001	-42.82 to -29.40	<0.0001
S2	c	NC	IgG1-b12	IgG1-b12	CDC	Wien-133		0.00	1.87	94.52 to 105.5	<0.0001		
S2	d	PC	IgG1-Campath-E430G	x	CDC	Wien-133	3	100.0	2.77			-105.1 to -94.89	<0.0001
S2	d	TI	IgG1-Campath-E430G-K439E	x	CDC	Wien-133	3	40.63	1.59	55.19 to 63.54	<0.0001	-44.81 to -36.46	<0.0001
S2	d	TI	IgG1-Campath	x	CDC	Wien-133	3	43.84	2.01	51.05 to 61.27	<0.0001	-48.95 to -38.73	<0.0001
S2	d	TI	IgG1-Campath-K439E	x	CDC	Wien-133	3	0.76	1.03	94.13 to 104.3	<0.0001	-5.872 to 4.348	0.9722
S2	d	NC	IgG1-b12	x	CDC	Wien-133	3	0.00	1.45	94.89 to 105.1	<0.0001		
S2	e	PC	IgG1-Campath-E430G	IgG1-11B8-E430G	FcyRIIa H131 activity	Raji	3	99.99	6.71			-110.3 to -89.67	<0.0001
S2	e	TI	IgG1-Campath-E430G-K439E	IgG1-b12	FcyRIIa H131 activity	Raji	3	2.46	0.48	87.21 to 107.8	<0.0001	-12.78 to 7.856	0.8932
S2	e	TI	IgG1-11B8-E430G-S440K	IgG1-b12	FcyRIIa H131 activity	Raji	3	9.01	0.98	80.66 to 101.3	<0.0001	-19.33 to 1.302	0.0904

S2	e	TI	IgG1-Campath-E430G-K439E	IgG1-11B8-E430G-S440K	FcyRIIa H131 activity	Raji	2	19.87	7.01	69.80 to 90.44	<0.0001	-30.19 to -9.555	0.0008
S2	e	NC	IgG1-b12	IgG1-b12	FcyRIIa H131 activity	Raji	3	0.00	0.30	89.67 to 110.3	<0.0001		
S2	f	PC	IgG1-Campath-E430G	IgG1-11B8-E430G	FcyRIIIa V158 activity	Raji	3	99.99	2.33			-107.7 to -92.29	<0.0001
S2	f	TI	IgG1-Campath-E430G-K439E	IgG1-b12	FcyRIIIa V158 activity	Raji	3	14.13	2.00	75.50 to 96.22	<0.0001	-21.84 to -6.428	0.0014
S2	f	TI	IgG1-11B8-E430G-S440K	IgG1-b12	FcyRIIIa V158 activity	Raji	3	110.50	5.14	-20.87 to -0.1465	0.0468	-118.2 to -102.8	<0.0001
S2	f	TI	IgG1-Campath-E430G-K439E	IgG1-11B8-E430G-S440K	FcyRIIIa V158 activity	Raji	2	94.10	4.45	-2.721 to 14.51	0.2079	-102.7 to -85.49	<0.0001
S2	f	NC	IgG1-b12	IgG1-b12	FcyRIIIa V158 activity	Raji	3	0.00	0.17	92.29 to 107.7	<0.0001		
S3	a	PC	IgG1-Campath-E430G	x	CDC	Wien-133	3	100.0	1.22			ND	ND
S3	a	TI	IgG1-Campath	x	CDC	Wien-133	3	39.06	1.02	59.43 to 64.92	<0.0001	ND	ND
S3	a	TI	IgG1-Campath-G237A-E430G	x	CDC	Wien-133	3	74.06	1.08	-0.2705 to 5.211	0.0763	ND	ND
S3	a	TI	IgG1-Campath-G236R-E430G	x	CDC	Wien-133	3	98.76	0.47	24.43 to 29.92	<0.0001	ND	ND
S3	b	PC	IgG1-Campath-E430G	IgG1-11B8-E430G	FcyRIIa H131 activity	Raji	3	99.99	6.71			-107.2 to -92.81	<0.0001
S3	b	TI	IgG1-Campath-RGE	IgG1-b12	FcyRIIa H131 activity	Raji	3	0.01	0.58	92.80 to 107.2	<0.0001	-7.188 to 7.173	>0.9999
S3	b	TI	IgG1-11B8-AGK	IgG1-b12	FcyRIIa H131 activity	Raji	3	-0.27	0.33	93.08 to 107.4	<0.0001	-6.917 to 7.445	0.9999
S3	b	TI	IgG1-Campath-RGE	IgG1-11B8-AGK	FcyRIIa H131 activity	Raji	3	1.20	0.82	91.61 to 106.0	<0.0001	-8.386 to 5.976	0.9652

S3	b	NC	IgG1-b12	IgG1-b12	FcyRIIa H131 activity	Raji	3	0.00	0.30	92.81 to 107.2	<0.0001		
S3	b	PC	IgG1-Campath-E430G	IgG1-11B8-E430G	FcyRIIIa V158 activity	Raji	3	99.99	2.33			-102.7 to -97.30	<0.0001
S3	b	TI	IgG1-Campath-RGE	IgG1-b12	FcyRIIIa V158 activity	Raji	3	1.03	0.98	96.27 to 101.7	<0.0001	-3.726 to 1.659	0.6487
S3	b	TI	IgG1-11B8-AGK	IgG1-b12	FcyRIIIa V158 activity	Raji	3	0.01	0.24	97.29 to 102.7	<0.0001	-2.701 to 2.683	>0.9999
S3	b	TI	IgG1-Campath-RGE	IgG1-11B8-AGK	FcyRIIIa V158 activity	Raji	3	0.17	0.16	97.13 to 102.5	<0.0001	-2.863 to 2.521	0.9990
S3	b	NC	IgG1-b12	IgG1-b12	FcyRIIIa V158 activity	Raji	3	0.00	0.17	97.30 to 102.7	<0.0001		
S3[*8]	c	PC	IgG1-Campath	IgG1-11B8	C1q binding	Wien-133	6	100.0	41.07			-10.43 to -0.7746	0.0132
S3	c	TI	IgG1-Campath-E430G	IgG1-11B8-E430G	C1q binding	Wien-133	6	1859	87.50	-108.2 to -88.82	<0.0001	-114.4 to -93.85	<0.0001
S3	c	TI	IgG1-Campath-RGE	IgG1-b12	C1q binding	Wien-133	6	8.93	5.36	0.2520 to 9.948	0.0202	-1.092 to 0.09230	0.0629
S3	c	TI	IgG1-11B8-E430G-S440K	IgG1-b12	C1q binding	Wien-133	6	16.07	5.36	-0.1480 to 9.548	0.0284	-1.492 to -0.3077	0.0016
S3	c	TI	IgG1-11B8-AGK	IgG1-b12	C1q binding	Wien-133	6	5.36	3.57	0.4746 to 10.13	0.0168	-0.7511 to 0.1511	0.1945
S3	c	TI	IgG1-Campath-RGE	IgG1-11B8-E430G-S440K	C1q binding	Wien-133	6	287.5	39.29	-15.58 to -5.424	<0.0001	-20.72 to -11.48	<0.0001
S3	c	TI	IgG1-Campath-RGE	IgG1-11B8-AGK	C1q binding	Wien-133	6	92.86	19.64	-4.160 to 4.960	>0.9999	-7.537 to -2.863	0.0007
S3	c	NC	IgG1-b12	IgG1-b12	C1q binding	Wien-133	6	0.00	3.57	0.7746 to 10.43	0.0132		
S6[*9]	d	PC	IgG1-DR5-01-E430G	IgG1-DR5-05-E430G	Apoptosis	COLO-205	9	-0.07	0.186			--91.16 to -107.4	<0.0001
S6	d	TI	IgG1-DR5-01	IgG1-DR5-05	Apoptosis	COLO-205	9	31.8	12.1	23.72 to 39.93	<0.0001	-59.33 to -75.54	<0.0001
S6	d	NC	IgG1-b12	IgG1-b12	Apoptosis	COLO-205	9	99.2	3.75	91.16 to 107.4	<0.0001		

S6*[9]	e	PC	IgG1-DR5-01-E430G	IgG1-DR5-05-E430G	Apoptosis	BxPC-3	9	32.19	3.99			-73.78 to -65.23	<0.0001
S6	e	TI	IgG1-DR5-01	IgG1-DR5-05	Apoptosis	BxPC-3	9	103.9	3.51	67.46 to 76.01	<0.0001	-2.043 to 6.506	0.3783
S6	e	NC	IgG1-b12	IgG1-b12	Apoptosis	BxPC-3	9	101.7	4.05	65.23 to 73.78	<0.0001		

*[1] Items: positive control (PC), negative control (NC) or test item (TI) used in comparisons. *[2] Number of independent experimental repeats, performed on separate days, using independently prepared antibody mixtures. *[3] Dose-response data from multiple experimental repeats were pooled, concentrations were log-transformed and the resulting AUC values were normalized relative to the positive control indicated in grey (100%) and negative control non-binding antibody IgG1-b12 (0%). *[4] 95% confidence interval of the difference between measured value (test item) and value measured for positive control antibody or antibody mixture. *[5] Adjusted p-value of one-way ANOVA analysis of comparison between test item and positive control using Dunnett's T3 multiple comparisons test. Analyses were performed using GraphPad Prism release 8.4.1. *[6] 95% confidence interval of the difference between test item and negative control values. *[7] Adjusted P-value calculated as described *[5], here for the difference between test item and negative control values. ND: not determined. *[8] Dunnett's T3 multiple comparisons test was used to address the different standard deviations between groups. *[9] Analysis of responses at single antibody concentration instead of dose-response curves.

Supplementary Table 4. Statistical analysis of Super Resolution Localization Microscopy data.

Fig.	Sub	Test item 1	n TI1*1	Test item 2	n TI2*1	Assay	Cells	Difference between test item means*2	95% CI of diff. *3	Adjusted P-value*4
4	c	IgG1-Campath-RGE-HA	47	IgG1-b12-RGE-HA	69	dSTORM	Wien-133	484.4	335.8 to 633.0	<0.0001
4	c	IgG1-Campath-RGE-HA +IgG1-11B8-AGK	17	IgG1-b12-RGE-HA	69	dSTORM	Wien-133	528.2	195.6 to 860.8	0.0003
4	c	IgG1-11B8-AGK-HA	41	IgG1-b12-RGE-HA	69	dSTORM	Wien-133	96.97	17.19 to 176.7	0.0028
4	c	IgG1-11B8-AGK-HA +IgG1-Campath-RGE	33	IgG1-b12-RGE-HA	69	dSTORM	Wien-133	159.7	91.15 to 228.2	<0.0001
4	c	IgG1-Campath-RGY-HA	14	IgG1-b12-RGE-HA	69	dSTORM	Wien-133	701.3	438.4 to 964.2	<0.0001
4	c	IgG1-Campath-RGE-HA	47	IgG1-Campath-RGY-HA	14	dSTORM	Wien-133	-216.9	-498.4 to 64.53	0.1554
4	c	IgG1-Campath-RGE-HA +IgG1-11B8-AGK	17	IgG1-Campath-RGY-HA	14	dSTORM	Wien-133	-173.1	-559.0 to 212.8	0.8383
4	c	IgG1-11B8-AGK-HA	41	IgG1-Campath-RGY-HA	14	dSTORM	Wien-133	-604.3	-868.7 to -340.0	<0.0001
4	c	IgG1-11B8-AGK-HA +IgG1-Campath-RGE	33	IgG1-Campath-RGY-HA	14	dSTORM	Wien-133	-541.6	-804.8 to -278.5	<0.0001
4	c	IgG1-Campath-RGE-HA	47	IgG1-Campath-RGE-HA +IgG1-11B8-AGK	17	dSTORM	Wien-133	-43.82	-392.6 to 305.0	>0.9999
4	c	IgG1-11B8-AGK-HA	41	IgG1-11B8-AGK-HA +IgG1-Campath-RGE	33	dSTORM	Wien-133	-62.70	-158.3 to 32.86	0.4067
4	d	IgG1-Campath-RGE-HA	47	IgG1-b12-RGE-HA	69	dSTORM	Wien-133	64.06	52.07 to 76.06	<0.0001
4	d	IgG1-Campath-RGE-HA +IgG1-11B8-AGK	17	IgG1-b12-RGE-HA	69	dSTORM	Wien-133	63.48	49.29 to 77.67	<0.0001
4	d	IgG1-11B8-AGK-HA	41	IgG1-b12-RGE-HA	69	dSTORM	Wien-133	38.47	25.45 to 51.49	<0.0001

4	d	IgG1-11B8-AGK-HA +IgG1-Campath-RGE	33	IgG1-b12-RGE-HA	69	dSTORM	Wien-133	45.55	31.78 to 59.32	<0.0001
4	d	IgG1-Campath-RGY-HA	14	IgG1-b12-RGE-HA	69	dSTORM	Wien-133	74.57	62.76 to 86.38	<0.0001
4	d	IgG1-Campath-RGE-HA	47	IgG1-Campath-RGY-HA	14	dSTORM	Wien-133	10.50	2.337 to 18.67	0.0014
4	d	IgG1-Campath-RGE-HA +IgG1-11B8-AGK	17	IgG1-Campath-RGY-HA	14	dSTORM	Wien-133	11.09	-0.4268 to 22.60	0.0342
4	d	IgG1-11B8-AGK-HA	41	IgG1-Campath-RGY-HA	14	dSTORM	Wien-133	36.10	26.45 to 45.74	<0.0001
4	d	IgG1-11B8-AGK-HA +IgG1-Campath-RGE	33	IgG1-Campath-RGY-HA	14	dSTORM	Wien-133	29.02	18.30 to 39.74	<0.0001
4	d	IgG1-Campath-RGE-HA	47	IgG1-Campath-RGE-HA +IgG1-11B8-AGK	17	dSTORM	Wien-133	0.5819	-11.05 to 12.21	>0.9999
4	d	IgG1-11B8-AGK-HA	41	IgG1-11B8-AGK-HA +IgG1-Campath-RGE	33	dSTORM	Wien-133	-7.076	-19.07 to 4.916	0.5710

*1 Number of Regions of Interest (ROI) analyzed for test item 1 (TI1) and test item 2 (TI2), respectively. *2 Difference between the Localizations (Fig. 4c) or averaged nearest neighbor distance (Fig. 4d) per region of interest measured for TI1 and TI2. *3 95% confidence interval of the difference between the localizations (Fig. 4c) or averaged nearest neighbor distance (Fig. 4d) per region of interest measured for TI1 and TI2. *5 Adjusted p-value of one-way ANOVA analysis after Welch correction for non-equal SD's, of difference between test items 1 and 2 using Dunnett's T3 multiple comparisons test.

Supplementary Table 5. Statistical analysis of Surface Plasmon Resonance data.

Fig.	Sub	Item[*1]	Antibody component 1	Assay	N[*2]	k_a	SD	k_d	SD	K_D	SD	95% CI of K_D diff. to PC[*3]	P-value[*4] of K_D comparison
5	a	PC	IgG1-Campath	FcRn binding	3	6.49E+05	5.69E+04	4.39E-02	1.18E-03	6.76E-08	6.21E-09		
5	a	TI	IgG1-Campath-RGE	FcRn binding	3	6.08E+05	5.98E+04	4.71E-02	1.48E-03	7.75E-08	8.00E-09	-6.334e-009 to 2.613e-008	0.1657
5	a	PC	IgG1-11B8	FcRn binding	3	8.72E+05	1.22E+05	4.89E-02	2.17E-03	5.61E-08	8.27E-09		
5	a	TI	IgG1-11B8-AGK	FcRn binding	3	1.09E+06	2.03E+05	6.26E-02	5.80E-03	5.75E-08	1.20E-08	-2.196e-008 to 2.476e-008	0.8759

*[1] Items: positive control (PC) or test item (TI) used in comparisons. [*2] N= Number of kinetics experiments, each consisting of a two-fold, eight-step dilution series of indicated IgG1 molecules (75 nM to 0.29 nM) injected onto sensorchips covalently linked to anti-His antibody loaded with His-tagged human FcRn/β2-microglobulin complex. [*3] . 95% confidence interval of the difference between the K_D of the TI compared and the PC. [*4] p-value of two-tailed T-test comparing K_D values of TI and PC.

Supplementary Table 6. Statistical analysis of Pharmacokinetics data.

Fig.	Sub	Item[*1]	Antibody component 1	Antibody component 2	Assay	Cells	N[*2]	Clearance rate[*3]	SD	95% CI of diff. to PC[*3]	Adjusted P-value*[4]
5	b	PC	IgG1-Campath	-	PK	N/A	3	28.8	6.7		
5	b	TI	IgG1-Campath-RGE	-	PK	N/A	3	34.6	7.3	-16.99 to 5.393	0.5862
5	b	PC	IgG1-11B8	-	PK	N/A	3	14	1.1		
5	b	TI	IgG1-11B8-AGK	-	PK	N/A	3	10.8	0.9	-14.39 to 7.993	0.9513
5	b	PC	IgG1-Campath	IgG1-11B8	PK	N/A	3	11.9	1.5		
5	b	TI	IgG1-Campath-RGE	IgG1-11B8-AGK	PK	N/A	3	11.8	1.9	-11.09 to 11.29	>0.9999

*[1] Items: positive control (PC) or test item (TI) used in comparisons. *[2] Number of mice. *[3] Clearance of a single antibody dose (500 µg) was monitored for three weeks and is expressed as Dose (D)*1000/area under the curve (ml/day/kg). *[4] 95% confidence interval of the difference between measured value (test item) and value measured for positive control antibody or antibody mixture. *[5] Adjusted p-value of one-way ANOVA analysis of comparison between test items and positive controls using Tukey's multiple comparisons test.

Supplementary Table 7. Statistical analysis of *in vivo* POC data.

Fig.	Sub	Test Item 1	n TI1[*1]	Test Item 2	n TI2[*2]	Assay	Cells	Difference between test item means[*2]	95% CI of diff. [*3]	Adjusted P-value*[4]
5	d	IgG1-b12-RGE+IgG1-b12-AGK	13	IgG1-b12-RGE+IgG1-11B8-AGK	13	Count	T cells	0.3481	-1.304 to 2.000	0.9970
5	d	IgG1-b12-RGE+IgG1-b12-AGK	13	IgG1-Campath-RGE+IgG1-b12-AGK	14	Count	T cells	-0.1800	-1.195 to 0.8346	0.9992
5	d	IgG1-b12-RGE+IgG1-b12-AGK	13	IgG1-Campath-RGE+IgG1-11B8-AGK	13	Count	T cells	-0.1328	-1.213 to 0.9475	>0.9999
5	d	IgG1-b12-RGE+IgG1-b12-AGK	13	IgG1-Campath-E430G+IgG1-11B8-E430G	14	Count	T cells	0.7980	-0.2140 to 1.810	0.1149
5	d	IgG1-b12-RGE+IgG1-11B8-AGK	13	IgG1-Campath-RGE+IgG1-b12-AGK	14	Count	T cells	-0.5282	-2.075 to 1.018	0.8993
5	d	IgG1-b12-RGE+IgG1-11B8-AGK	13	IgG1-Campath-RGE+IgG1-11B8-AGK	13	Count	T cells	-0.4809	-2.057 to 1.095	0.9525
5	d	IgG1-b12-RGE+IgG1-11B8-AGK	13	IgG1-Campath-E430G+IgG1-11B8-E430G	14	Count	T cells	0.4499	-1.090 to 1.989	0.9572
5	d	IgG1-Campath-RGE+IgG1-b12-AGK	14	IgG1-Campath-RGE+IgG1-11B8-AGK	13	Count	T cells	0.04722	-0.7508 to 0.8452	>0.9999
5	d	IgG1-Campath-RGE+IgG1-b12-AGK	14	IgG1-Campath-E430G+IgG1-11B8-E430G	14	Count	T cells	0.9780	0.3204 to 1.636	0.0004
5	d	IgG1-Campath-RGE+IgG1-11B8-AGK	13	IgG1-Campath-E430G+IgG1-11B8-E430G	14	Count	T cells	0.9308	0.1413 to 1.720	0.0059
5	d	IgG1-b12-RGE+IgG1-b12-AGK	13	IgG1-b12-RGE+IgG1-11B8-AGK	13	Count	B cells	0.4251	-1.119 to 1.969	0.9830
5	d	IgG1-b12-RGE+IgG1-b12-AGK	13	IgG1-Campath-RGE+IgG1-b12-AGK	14	Count	B cells	-0.05129	-1.329 to 1.226	>0.9999
5	d	IgG1-b12-RGE+IgG1-b12-AGK	13	IgG1-Campath-RGE+IgG1-11B8-AGK	13	Count	B cells	1.582	0.3326 to 2.832	0.0033
5	d	IgG1-b12-RGE+IgG1-b12-AGK	13	IgG1-Campath-E430G+IgG1-11B8-E430G	14	Count	B cells	1.806	0.6105 to 3.001	0.0007
5	d	IgG1-b12-RGE+IgG1-11B8-AGK	13	IgG1-Campath-RGE+IgG1-b12-AGK	14	Count	B cells	-0.4764	-1.804 to 0.8512	0.9002
5	d	IgG1-b12-RGE+IgG1-11B8-AGK	13	IgG1-Campath-RGE+IgG1-11B8-AGK	13	Count	B cells	1.157	-0.1453 to 2.460	0.0569
5	d	IgG1-b12-RGE+IgG1-11B8-AGK	13	IgG1-Campath-E430G+IgG1-11B8-E430G	14	Count	B cells	1.380	0.1114 to 2.649	0.0136

5	d	IgG1-Campath-RGE+IgG1-b12-AGK	14	IgG1-Campath-RGE+IgG1-11B8-AGK	13	Count	B cells	1.634	0.7669 to 2.500	<0.0001
5	d	IgG1-Campath-RGE+IgG1-b12-AGK	14	IgG1-Campath-E430G+IgG1-11B8-E430G	14	Count	B cells	1.857	1.093 to 2.621	<0.0001
5	d	IgG1-Campath-RGE+IgG1-11B8-AGK	13	IgG1-Campath-E430G+IgG1-11B8-E430G	14	Count	B cells	0.2232	-0.4618 to 0.9081	0.9411
5	d	IgG1-b12-RGE+IgG1-b12-AGK	13	IgG1-b12-RGE+IgG1-11B8-AGK	13	MFI C3	B cells	-0.2501	-0.6074 to 0.1073	0.2095
5	d	IgG1-b12-RGE+IgG1-b12-AGK	13	IgG1-Campath-RGE+IgG1-b12-AGK	14	MFI C3	B cells	-0.02591	-0.2311 to 0.1793	>0.9999
5	d	IgG1-b12-RGE+IgG1-b12-AGK	13	IgG1-Campath-RGE+IgG1-11B8-AGK	13	MFI C3	B cells	-1.176	-1.422 to -0.9306	<0.0001
5	d	IgG1-b12-RGE+IgG1-b12-AGK	13	IgG1-Campath-E430G+IgG1-11B8-E430G	14	MFI C3	B cells	-1.184	-1.474 to -0.8937	<0.0001
5	d	IgG1-b12-RGE+IgG1-11B8-AGK	13	IgG1-Campath-RGE+IgG1-b12-AGK	14	MFI C3	B cells	0.2242	-0.1199 to 0.5682	0.2572
5	d	IgG1-b12-RGE+IgG1-11B8-AGK	13	IgG1-Campath-RGE+IgG1-11B8-AGK	13	MFI C3	B cells	-0.9260	-1.287 to -0.5646	<0.0001
5	d	IgG1-b12-RGE+IgG1-11B8-AGK	13	IgG1-Campath-E430G+IgG1-11B8-E430G	14	MFI C3	B cells	-0.9336	-1.318 to -0.5491	<0.0001
5	d	IgG1-Campath-RGE+IgG1-b12-AGK	14	IgG1-Campath-RGE+IgG1-11B8-AGK	13	MFI C3	B cells	-1.150	-1.363 to -0.9369	<0.0001
5	d	IgG1-Campath-RGE+IgG1-b12-AGK	14	IgG1-Campath-E430G+IgG1-11B8-E430G	14	MFI C3	B cells	-1.158	-1.425 to -0.8902	<0.0001
5	d	IgG1-Campath-RGE+IgG1-11B8-AGK	13	IgG1-Campath-E430G+IgG1-11B8-E430G	14	MFI C3	B cells	-0.007553	-0.3009 to 0.2858	>0.9999
5	e	IgG1-b12-RGE+IgG1-b12-AGK	14	IgG1-b12-RGE+IgG1-11B8-AGK	14	Count	T cells	-0.04237	-0.7294 to 0.6447	-0.04237
5	e	IgG1-b12-RGE+IgG1-b12-AGK	14	IgG1-Campath-RGE+IgG1-b12-AGK	13	Count	T cells	0.1629	-0.5321 to 0.8579	0.1629
5	e	IgG1-b12-RGE+IgG1-b12-AGK	14	IgG1-Campath-RGE+IgG1-11B8-AGK	15	Count	T cells	0.2563	-0.5621 to 1.075	0.2563
5	e	IgG1-b12-RGE+IgG1-b12-AGK	14	IgG1-Campath-E430G+IgG1-11B8-E430G	11	Count	T cells	1.373	0.5628 to 2.183	1.373
5	e	IgG1-b12-RGE+IgG1-11B8-AGK	14	IgG1-Campath-RGE+IgG1-b12-AGK	13	Count	T cells	0.2052	-0.4744 to 0.8849	0.2052
5	e	IgG1-b12-RGE+IgG1-11B8-AGK	14	IgG1-Campath-RGE+IgG1-11B8-AGK	15	Count	T cells	0.2986	-0.5100 to 1.107	0.2986
5	e	IgG1-b12-RGE+IgG1-11B8-AGK	14	IgG1-Campath-E430G+IgG1-11B8-E430G	11	Count	T cells	1.415	0.6139 to 2.217	1.415
5	e	IgG1-Campath-RGE+IgG1-b12-AGK	13	IgG1-Campath-RGE+IgG1-11B8-AGK	15	Count	T cells	0.09341	-0.7197 to 0.9065	0.09341

5	e	IgG1-Campath-RGE+IgG1-b12-AGK	13	IgG1-Campath-E430G+IgG1-11B8-E430G	11	Count	T cells	1.210	0.4039 to 2.017	1.210
5	e	IgG1-Campath-RGE+IgG1-11B8-AGK	15	IgG1-Campath-E430G+IgG1-11B8-E430G	11	Count	T cells	1.117	0.2133 to 2.020	1.117
5	e	IgG1-b12-RGE+IgG1-b12-AGK	14	IgG1-b12-RGE+IgG1-11B8-AGK	14	Count	B cells	0.4355	-0.04707 to 0.9182	0.0540
5	e	IgG1-b12-RGE+IgG1-b12-AGK	14	IgG1-Campath-RGE+IgG1-b12-AGK	13	Count	B cells	0.2396	-0.2852 to 0.7644	0.7231
5	e	IgG1-b12-RGE+IgG1-b12-AGK	14	IgG1-Campath-RGE+IgG1-11B8-AGK	15	Count	B cells	1.927	1.031 to 2.824	<0.0001
5	e	IgG1-b12-RGE+IgG1-b12-AGK	14	IgG1-Campath-E430G+IgG1-11B8-E430G	11	Count	B cells	1.971	1.513 to 2.429	<0.0001
5	e	IgG1-b12-RGE+IgG1-11B8-AGK	14	IgG1-Campath-RGE+IgG1-b12-AGK	13	Count	B cells	-0.1959	-0.7600 to 0.3682	0.9274
5	e	IgG1-b12-RGE+IgG1-11B8-AGK	14	IgG1-Campath-RGE+IgG1-11B8-AGK	15	Count	B cells	1.492	0.5802 to 2.403	0.0002
5	e	IgG1-b12-RGE+IgG1-11B8-AGK	14	IgG1-Campath-E430G+IgG1-11B8-E430G	11	Count	B cells	1.536	1.029 to 2.042	<0.0001
5	e	IgG1-Campath-RGE+IgG1-b12-AGK	13	IgG1-Campath-RGE+IgG1-11B8-AGK	15	Count	B cells	1.688	0.7619 to 2.613	<0.0001
5	e	IgG1-Campath-RGE+IgG1-b12-AGK	13	IgG1-Campath-E430G+IgG1-11B8-E430G	11	Count	B cells	1.731	1.189 to 2.274	<0.0001
5	e	IgG1-Campath-RGE+IgG1-11B8-AGK	15	IgG1-Campath-E430G+IgG1-11B8-E430G	11	Count	B cells	0.04380	-0.8638 to 0.9514	>0.9999
5	e	IgG1-b12-RGE+IgG1-b12-AGK	14	IgG1-b12-RGE+IgG1-11B8-AGK	14	MFI C3	B cells	-0.7932	-0.9428 to -0.6436	<0.0001
5	e	IgG1-b12-RGE+IgG1-b12-AGK	14	IgG1-Campath-RGE+IgG1-b12-AGK	13	MFI C3	B cells	-0.01924	-0.1405 to 0.1020	0.9998
5	e	IgG1-b12-RGE+IgG1-b12-AGK	14	IgG1-Campath-RGE+IgG1-11B8-AGK	15	MFI C3	B cells	-1.035	-1.444 to -0.6263	<0.0001
5	e	IgG1-b12-RGE+IgG1-b12-AGK	14	IgG1-Campath-E430G+IgG1-11B8-E430G	11	MFI C3	B cells	-0.9064	-1.400 to -0.4132	0.0002
5	e	IgG1-b12-RGE+IgG1-11B8-AGK	14	IgG1-Campath-RGE+IgG1-b12-AGK	13	MFI C3	B cells	0.7740	0.6215 to 0.9265	<0.0001
5	e	IgG1-b12-RGE+IgG1-11B8-AGK	14	IgG1-Campath-RGE+IgG1-11B8-AGK	15	MFI C3	B cells	-0.2420	-0.6554 to 0.1714	0.3370
5	e	IgG1-b12-RGE+IgG1-11B8-AGK	14	IgG1-Campath-E430G+IgG1-11B8-E430G	11	MFI C3	B cells	-0.1132	-0.6071 to 0.3808	0.9870
5	e	IgG1-Campath-RGE+IgG1-b12-AGK	13	IgG1-Campath-RGE+IgG1-11B8-AGK	15	MFI C3	B cells	-1.016	-1.426 to -0.6057	<0.0001
5	e	IgG1-Campath-RGE+IgG1-b12-AGK	13	IgG1-Campath-E430G+IgG1-11B8-E430G	11	MFI C3	B cells	-0.8872	-1.382 to -0.3928	0.0003

5	e	IgG1-Campath-RGE+IgG1-11B8-AGK	15	IgG1-Campath-E430G+IgG1-11B8-E430G	11	MFI C3	B cells	0.1289	-0.4380 to 0.6957	0.9943
5	f	IgG1-b12-RGE+IgG1-b12-AGK	14	IgG1-b12-RGE+IgG1-11B8-AGK	14	Count	T cells	-0.04899	-0.5160 to 0.4180	>0.9999
5	f	IgG1-b12-RGE+IgG1-b12-AGK	14	IgG1-Campath-RGE+IgG1-b12-AGK	13	Count	T cells	-0.02818	-0.4344 to 0.3780	>0.9999
5	f	IgG1-b12-RGE+IgG1-b12-AGK	14	IgG1-Campath-RGE+IgG1-11B8-AGK	15	Count	T cells	0.02322	-0.3573 to 0.4037	>0.9999
5	f	IgG1-b12-RGE+IgG1-b12-AGK	14	IgG1-Campath-E430G+IgG1-11B8-E430G	11	Count	T cells	2.086	1.519 to 2.653	<0.0001
5	f	IgG1-b12-RGE+IgG1-11B8-AGK	14	IgG1-Campath-RGE+IgG1-b12-AGK	13	Count	T cells	0.02081	-0.4268 to 0.4684	>0.9999
5	f	IgG1-b12-RGE+IgG1-11B8-AGK	14	IgG1-Campath-RGE+IgG1-11B8-AGK	15	Count	T cells	0.07221	-0.3525 to 0.4970	0.9996
5	f	IgG1-b12-RGE+IgG1-11B8-AGK	14	IgG1-Campath-E430G+IgG1-11B8-E430G	11	Count	T cells	2.135	1.545 to 2.725	<0.0001
5	f	IgG1-Campath-RGE+IgG1-b12-AGK	13	IgG1-Campath-RGE+IgG1-11B8-AGK	15	Count	T cells	0.05140	-0.3001 to 0.4029	0.9999
5	f	IgG1-Campath-RGE+IgG1-b12-AGK	13	IgG1-Campath-E430G+IgG1-11B8-E430G	11	Count	T cells	2.114	1.563 to 2.665	<0.0001
5	f	IgG1-Campath-RGE+IgG1-11B8-AGK	15	IgG1-Campath-E430G+IgG1-11B8-E430G	11	Count	T cells	2.063	1.525 to 2.600	<0.0001
5	f	IgG1-b12-RGE+IgG1-b12-AGK	14	IgG1-b12-RGE+IgG1-11B8-AGK	14	Count	B cells	0.7360	0.4717 to 1.000	<0.0001
5	f	IgG1-b12-RGE+IgG1-b12-AGK	14	IgG1-Campath-RGE+IgG1-b12-AGK	13	Count	B cells	0.05906	-0.1673 to 0.2854	0.9847
5	f	IgG1-b12-RGE+IgG1-b12-AGK	14	IgG1-Campath-RGE+IgG1-11B8-AGK	15	Count	B cells	1.154	0.8807 to 1.428	<0.0001
5	f	IgG1-b12-RGE+IgG1-b12-AGK	14	IgG1-Campath-E430G+IgG1-11B8-E430G	11	Count	B cells	1.534	1.176 to 1.891	<0.0001
5	f	IgG1-b12-RGE+IgG1-11B8-AGK	14	IgG1-Campath-RGE+IgG1-b12-AGK	13	Count	B cells	-0.6769	-0.9777 to -0.3762	<0.0001
5	f	IgG1-b12-RGE+IgG1-11B8-AGK	14	IgG1-Campath-RGE+IgG1-11B8-AGK	15	Count	B cells	0.4183	0.08534 to 0.7512	0.0028
5	f	IgG1-b12-RGE+IgG1-11B8-AGK	14	IgG1-Campath-E430G+IgG1-11B8-E430G	11	Count	B cells	0.7976	0.3999 to 1.195	<0.0001
5	f	IgG1-Campath-RGE+IgG1-b12-AGK	13	IgG1-Campath-RGE+IgG1-11B8-AGK	15	Count	B cells	1.095	0.7870 to 1.403	<0.0001
5	f	IgG1-Campath-RGE+IgG1-b12-AGK	13	IgG1-Campath-E430G+IgG1-11B8-E430G	11	Count	B cells	1.474	1.095 to 1.854	<0.0001
5	f	IgG1-Campath-RGE+IgG1-11B8-AGK	15	IgG1-Campath-E430G+IgG1-11B8-E430G	11	Count	B cells	0.3793	-0.02143 to 0.7800	0.0383

5	f	IgG1-b12-RGE+IgG1-b12-AGK	14	IgG1-b12-RGE+IgG1-11B8-AGK	14	MFI C3	B cells	-0.3280	-0.4378 to -0.2182	<0.0001
5	f	IgG1-b12-RGE+IgG1-b12-AGK	14	IgG1-Campath-RGE+IgG1-b12-AGK	13	MFI C3	B cells	0.005852	-0.1197 to 0.1314	>0.9999
5	f	IgG1-b12-RGE+IgG1-b12-AGK	14	IgG1-Campath-RGE+IgG1-11B8-AGK	15	MFI C3	B cells	-0.6281	-0.7334 to -0.5229	<0.0001
5	f	IgG1-b12-RGE+IgG1-b12-AGK	14	IgG1-Campath-E430G+IgG1-11B8-E430G	11	MFI C3	B cells	-0.5590	-0.7597 to -0.3583	<0.0001
5	f	IgG1-b12-RGE+IgG1-11B8-AGK	14	IgG1-Campath-RGE+IgG1-b12-AGK	13	MFI C3	B cells	0.3339	0.2101 to 0.4576	<0.0001
5	f	IgG1-b12-RGE+IgG1-11B8-AGK	14	IgG1-Campath-RGE+IgG1-11B8-AGK	15	MFI C3	B cells	-0.3001	-0.4025 to -0.1977	<0.0001
5	f	IgG1-b12-RGE+IgG1-11B8-AGK	14	IgG1-Campath-E430G+IgG1-11B8-E430G	11	MFI C3	B cells	-0.2310	-0.4305 to -0.03145	0.0076
5	f	IgG1-Campath-RGE+IgG1-b12-AGK	13	IgG1-Campath-RGE+IgG1-11B8-AGK	15	MFI C3	B cells	-0.6340	-0.7544 to -0.5135	<0.0001
5	f	IgG1-Campath-RGE+IgG1-b12-AGK	13	IgG1-Campath-E430G+IgG1-11B8-E430G	11	MFI C3	B cells	-0.5648	-0.7704 to -0.3593	<0.0001
5	f	IgG1-Campath-RGE+IgG1-11B8-AGK	15	IgG1-Campath-E430G+IgG1-11B8-E430G	11	MFI C3	B cells	0.06913	-0.1291 to 0.2674	0.9009
S6	a	IgG1-b12-RGE+IgG1-b12-AGK	13	IgG1-b12-RGE+IgG1-11B8-AGK	13	MFI C3	T cells	-0.04993	-0.2821 to 0.1823	0.9973
S6	a	IgG1-b12-RGE+IgG1-b12-AGK	13	IgG1-Campath-RGE+IgG1-b12-AGK	14	MFI C3	T cells	-0.09290	-0.3249 to 0.1391	0.8519
S6	a	IgG1-b12-RGE+IgG1-b12-AGK	13	IgG1-Campath-RGE+IgG1-11B8-AGK	13	MFI C3	T cells	-0.1523	-0.4129 to 0.1084	0.4255
S6	a	IgG1-b12-RGE+IgG1-b12-AGK	13	IgG1-Campath-E430G+IgG1-11B8-E430G	14	MFI C3	T cells	-0.9549	-1.173 to -0.7370	<0.0001
S6	a	IgG1-b12-RGE+IgG1-11B8-AGK	13	IgG1-Campath-RGE+IgG1-b12-AGK	14	MFI C3	T cells	-0.04298	-0.2859 to 0.1999	0.9995
S6	a	IgG1-b12-RGE+IgG1-11B8-AGK	13	IgG1-Campath-RGE+IgG1-11B8-AGK	13	MFI C3	T cells	-0.1023	-0.3716 to 0.1669	0.8792
S6	a	IgG1-b12-RGE+IgG1-11B8-AGK	13	IgG1-Campath-E430G+IgG1-11B8-E430G	14	MFI C3	T cells	-0.9050	-1.135 to -0.6745	<0.0001
S6	a	IgG1-Campath-RGE+IgG1-b12-AGK	14	IgG1-Campath-RGE+IgG1-11B8-AGK	13	MFI C3	T cells	-0.05935	-0.3281 to 0.2094	0.9967
S6	a	IgG1-Campath-RGE+IgG1-b12-AGK	14	IgG1-Campath-E430G+IgG1-11B8-E430G	14	MFI C3	T cells	-0.8620	-1.091 to -0.6326	<0.0001
S6	a	IgG1-Campath-RGE+IgG1-11B8-AGK	13	IgG1-Campath-E430G+IgG1-11B8-E430G	14	MFI C3	T cells	-0.8027	-1.060 to -0.5449	<0.0001
S6	b	IgG1-b12-RGE+IgG1-b12-AGK	14	IgG1-b12-RGE+IgG1-11B8-AGK	14	MFI C3	T cells PL	-0.01401	-0.1968 to 0.1688	>0.9999

S6	b	IgG1-b12-RGE+IgG1-b12-AGK	14	IgG1-Campath-RGE+IgG1-b12-AGK	13	MFI C3	T cells PL	-0.009255	-0.1937 to 0.1752	>0.9999
S6	b	IgG1-b12-RGE+IgG1-b12-AGK	14	IgG1-Campath-RGE+IgG1-11B8-AGK	15	MFI C3	T cells PL	0.02962	-0.1471 to 0.2064	0.9996
S6	b	IgG1-b12-RGE+IgG1-b12-AGK	14	IgG1-Campath-E430G+IgG1-11B8-E430G	11	MFI C3	T cells PL	-0.6556	-0.8891 to -0.4222	<0.0001
S6	b	IgG1-b12-RGE+IgG1-11B8-AGK	14	IgG1-Campath-RGE+IgG1-b12-AGK	13	MFI C3	T cells PL	0.004756	-0.1533 to 0.1628	>0.9999
S6	b	IgG1-b12-RGE+IgG1-11B8-AGK	14	IgG1-Campath-RGE+IgG1-11B8-AGK	15	MFI C3	T cells PL	0.04363	-0.1039 to 0.1911	0.9745
S6	b	IgG1-b12-RGE+IgG1-11B8-AGK	14	IgG1-Campath-E430G+IgG1-11B8-E430G	11	MFI C3	T cells PL	-0.6416	-0.8610 to -0.4223	<0.0001
S6	b	IgG1-Campath-RGE+IgG1-b12-AGK	13	IgG1-Campath-RGE+IgG1-11B8-AGK	15	MFI C3	T cells PL	0.03887	-0.1112 to 0.1890	0.9894
S6	b	IgG1-Campath-RGE+IgG1-b12-AGK	13	IgG1-Campath-E430G+IgG1-11B8-E430G	11	MFI C3	T cells PL	-0.6464	-0.8673 to -0.4255	<0.0001
S6	b	IgG1-Campath-RGE+IgG1-11B8-AGK	15	IgG1-Campath-E430G+IgG1-11B8-E430G	11	MFI C3	T cells PL	-0.6853	-0.9004 to -0.4701	<0.0001
S6	b	IgG1-b12-RGE+IgG1-b12-AGK	14	IgG1-b12-RGE+IgG1-11B8-AGK	14	MFI C3	T cells BL	0.03059	-0.05229 to 0.1135	0.9016
S6	b	IgG1-b12-RGE+IgG1-b12-AGK	14	IgG1-Campath-RGE+IgG1-b12-AGK	13	MFI C3	T cells BL	-0.04073	-0.09868 to 0.01722	0.2122
S6	b	IgG1-b12-RGE+IgG1-b12-AGK	14	IgG1-Campath-RGE+IgG1-11B8-AGK	15	MFI C3	T cells BL	-0.05096	-0.1087 to 0.006745	0.0608
S6	b	IgG1-b12-RGE+IgG1-b12-AGK	14	IgG1-Campath-E430G+IgG1-11B8-E430G	11	MFI C3	T cells BL	-0.4119	-0.6559 to -0.1678	0.0003
S6	b	IgG1-b12-RGE+IgG1-11B8-AGK	14	IgG1-Campath-RGE+IgG1-b12-AGK	13	MFI C3	T cells BL	-0.07132	-0.1467 to 0.004082	0.0376
S6	b	IgG1-b12-RGE+IgG1-11B8-AGK	14	IgG1-Campath-RGE+IgG1-11B8-AGK	15	MFI C3	T cells BL	-0.08155	-0.1568 to -0.006341	0.0131
S6	b	IgG1-b12-RGE+IgG1-11B8-AGK	14	IgG1-Campath-E430G+IgG1-11B8-E430G	11	MFI C3	T cells BL	-0.4425	-0.6884 to -0.1965	0.0001
S6	b	IgG1-Campath-RGE+IgG1-b12-AGK	13	IgG1-Campath-RGE+IgG1-11B8-AGK	15	MFI C3	T cells BL	-0.01023	-0.05081 to 0.03035	0.9917
S6	b	IgG1-Campath-RGE+IgG1-b12-AGK	13	IgG1-Campath-E430G+IgG1-11B8-E430G	11	MFI C3	T cells BL	-0.3711	-0.6149 to -0.1274	0.0008
S6	b	IgG1-Campath-RGE+IgG1-11B8-AGK	15	IgG1-Campath-E430G+IgG1-11B8-E430G	11	MFI C3	T cells BL	-0.3609	-0.6046 to -0.1172	0.0011

*[1] Number of mice treated with test item 1 (TI1). *[2] Number of mice treated with test item 2 (TI2). *[3] Difference between the log transformed values measured for TI1 and TI2. *[4] 95% confidence interval of the difference between the log values measured for TI1

and TI2. *[5] Adjusted p-value of one-way ANOVA analysis after Welch correction for non-equal SD's, of comparison between log values measured for TI1 and TI2 using Dunnett's T3 multiple comparisons test.

Supplementary Table 8. Antibodies and reagents used for *in vivo* proof-of-concept studies.

Target	Label	Company	Clone	Cat. No.	Dilution	Panel
hCD45	BV785	BioLegend	HI30	304048	100x	T- and B-cell depletion, and C3 fixation
hCD22	PE	BD Biosciences	S-HCL-1	337899	20x	
hCD19	PE-CF594	BD Biosciences	HIB19	562294	200x	
hCD2	APC	eBioscience	RPA-2.10	17-0029-42	200x	
hCD3	eFluor405	eBioscience	OKT3	48-0037-42	200x	
mC3	FITC	Cedarlane	RmC11H9	C7503F	100x	
Mouse Fc block		BD Biosciences		553142	200x	
Human Fc block		BD Biosciences		564220	200x	
Fixable Viability staining	BV510	BD Biosciences		564406	2000x	
hCD3	eFluor405	eBioscience	OKT3	48-0037-42	100x	Quantification level of humanization (NSG-HIS model)
hCD14	FITC	BD Biosciences	MφP-9	345784	50x	
hCD19	PE	Beckman Coulter	J3-119	A07769	50x	
hCD11c	PE-Cy7	BioLegend	Bu15	337215	400x	
hCD33	APC	BD Biosciences	WM53	551378	400x	
hCD56	PerCP-eFluor710	eBioscience	CMSSB	46-0567-41	50x	
hCD45	BV785	BioLegend	HI30	304048	40x	
mCD45	APC-eFluor780	eBioscience	30-F11	47-0451-82	100x	

Mouse Fc block		BD Biosciences		553142	100x	
Human Fc block		BD Biosciences		564220	100x	
Viability dye FVS700	BV700	BD Biosciences		564997	1000x	

Supplementary Table 9. Population characteristics of PBMCs derived from patients with chronic lymphocytic leukemia

Patient #	Primary diagnosis	Age at collection	Gender	Race	Ethnicity	Overall clinical stage	Disease status	Overall treatment (Tx) status	Treatment (Tx) status: chemo/hormone treatment	Treatment (Tx) status: radiation	Treatment (Tx) notes
1	Chronic Lymphocytic leukemia (CLL)	75	Female	White	Non-Hispanic	0	Progressive	Pre Tx	Pre Tx	Not applicable	-
2	Chronic Lymphocytic leukemia (CLL)	76	Female	White	Non-Hispanic	0	-	Refractory	Post Tx	Pre Tx	Post Tx with Rituxan and fludarabine (excellent response, complete remission)
3	Chronic Lymphocytic leukemia (CLL)	91	Male	White	Non-Hispanic	0	-	Pre Tx	Pre Tx	Pre Tx	-
