# Supplementary material for: Logic-gated antibody pairs that selectively act on cells co-expressing two antigens
Source: Nat Biotechnol. 2022 Jul 25;40(10):1509–19. doi: 10.1038/s41587-022-01384-1 (PMC9546771; doi:10.1038/s41587-022-01384-1)
Supplement: Supplementary file 2 — Reporting Summary. [file 41587_2022_1384_MOESM2_ESM.pdf]

nature research

Corresponding author(s): Rob. N. de Jong

Last updated by author(s): Jul 7, 2022

Reporting Summary

Nature Research wishes to improve the reproducibility of the work that we publish. This form provides structure for consistency and transparency in reporting. For further information on Nature Research policies, see our Editorial Policies and the Editorial Policy Checklist.

Statistics

For all statistical analyses, confirm that the following items are present in the figure legend, table legend, main text, or Methods section.

n/a	Confirmed	
☐	☒	The exact sample size (*n*) for each experimental group/condition, given as a discrete number and unit of measurement
☐	☒	A statement on whether measurements were taken from distinct samples or whether the same sample was measured repeatedly
☐	☒	The statistical test(s) used AND whether they are one- or two-sided<br>*Only common tests should be described solely by name; describe more complex techniques in the Methods section.*
☒	☐	A description of all covariates tested
☐	☒	A description of any assumptions or corrections, such as tests of normality and adjustment for multiple comparisons
☐	☒	A full description of the statistical parameters including central tendency (e.g. means) or other basic estimates (e.g. regression coefficient) AND variation (e.g. standard deviation) or associated estimates of uncertainty (e.g. confidence intervals)
☐	☒	For null hypothesis testing, the test statistic (e.g. *F*, *t*, *r*) with confidence intervals, effect sizes, degrees of freedom and *P* value noted<br>*Give P values as exact values whenever suitable.*
☒	☐	For Bayesian analysis, information on the choice of priors and Markov chain Monte Carlo settings
☒	☐	For hierarchical and complex designs, identification of the appropriate level for tests and full reporting of outcomes
☒	☐	Estimates of effect sizes (e.g. Cohen's *d*, Pearson's *r*), indicating how they were calculated

Our web collection on statistics for biologists contains articles on many of the points above.

Software and code

Policy information about availability of computer code

Data collection	IntelliCyt iQue Screener flow cytometer (Sartorius), LSRFortessa flow cytometer (BD Biosciences), Microplate reader (BioTek), EnVision Multilabel Reader (PerkinElmer), MesoScale Diagnostics QuickPlex SQ 120 (MSD), Biacore 8K SPR system (Cytiva).
Data analysis	LSR Fortesssa flow cytometry data was processed using FACSDiva v8 and v9.0 software (BD). Sartorius iQue flow cytometry data was processed using iQue ForeCyt v6.2 and v8.1 software (Sartorius). Flow cytometry data were analyzed using FlowJo V10 software (BD). Graphs were plotted and analyzed using GraphPad Prism 8.0 (DotMatics). EnVision data was processed using EnVision Workstation 1.13.3009.1401 software (Perkin Elmer). U-PLEX ProInflam data was processed using MesoScale Diagnostics Discovery Workbench v4.0. BioTek absorbance data was analyzed using BioTek Gen5 V1.04.5. BaGoL analysis was performed with the MATLAB implementation of BaGoL included with smite (https://github.com/LidkeLab/smite/) version 0.1.0 at the UNM Center for High Performance Computing (CARC) using MATLAB R2019b; post analysis of NN distance per ROI was computed with locally written MATLAB R2019b software using algorithm "knnsearch".

For manuscripts utilizing custom algorithms or software that are central to the research but not yet described in published literature, software must be made available to editors and reviewers. We strongly encourage code deposition in a community repository (e.g. GitHub). See the Nature Research guidelines for submitting code & software for further information.

Data

Policy information about availability of data

All manuscripts must include a data availability statement. This statement should provide the following information, where applicable:

- Accession codes, unique identifiers, or web links for publicly available datasets
- A list of figures that have associated raw data
- A description of any restrictions on data availability

Sequences of the variable domains and constant domains of recombinant antibodies tested herein were described in patent WO 2019/211472. The datasets generated during and/or analyzed during the current study are available from the corresponding author on reasonable request.

Field-specific reporting

Please select the one below that is the best fit for your research. If you are not sure, read the appropriate sections before making your selection.

☒ Life sciences ☐ Behavioural & social sciences ☐ Ecological, evolutionary & environmental sciences

For a reference copy of the document with all sections, see nature.com/documents/nr-reporting-summary-flat.pdf

Life sciences study design

All studies must disclose on these points even when the disclosure is negative.

Sample size	All data shown are representative of at least three independent replicate experiments or three individual human donors tested. No sample size calculation was performed prior to execution of experiments; post-analysis of experiments demonstrated which group comparisons were sufficiently powered to draw statistically significant conclusions (see Supplementary Tables 3-7). For in vivo studies, group sizes were estimated based on previous experience with similar but not identical experimental settings.
Data exclusions	Whole blood donor samples from which insufficient target/effector cells could be recovered were excluded from analysis. All other samples were included. No mice were excluded from analysis, but lipid contamination of some samples precluded FACS analysis.
Replication	All data shown are representative of at least three independent replicate experiments or at least three individual human donors tested. All attempts at replication were successful.
Randomization	For in vivo studies, NSG mice were randomly assigned to experimental groups. NSG-HIS mice were randomized into different treatment groups based on the percentage of circulating B and T cells of the total hCD45+ population. For samples used in in vitro studies, randomization was not applicable, as sufficient donor material was available to test each treatment group on each donor sample.
Blinding	For in vivo studies, the investigators who directly performed the study were blinded to the group allocation during execution of the in vivo experiment and the ex vivo analysis. For in vitro studies, group allocation was not applicable, as cells from each donor were exposed to all test items.

Reporting for specific materials, systems and methods

We require information from authors about some types of materials, experimental systems and methods used in many studies. Here, indicate whether each material, system or method listed is relevant to your study. If you are not sure if a list item applies to your research, read the appropriate section before selecting a response.

Materials & experimental systems

n/a	Involved in the study	
☐	☒	Antibodies
☐	☒	Eukaryotic cell lines
☒	☐	Palaeontology and archaeology
☐	☒	Animals and other organisms
☐	☒	Human research participants
☒	☐	Clinical data
☒	☐	Dual use research of concern

Methods

n/a	Involved in the study	
☒	☐	ChIP-seq
☐	☒	Flow cytometry
☒	☐	MRI-based neuroimaging

Antibodies

Antibodies used	Sequences of the variable domains and constant domains of recombinant antibodies tested herein were described in patent WO 2019/211472.<br><br>Commercially available antibodies used:

1. PE-Cy7 Mouse Anti-Human CD14 (clone M5E2, BD, Cat#557742, lots 0351935, 0188155, dilution 1:50)
2. PE Mouse Anti-Human CD11b (clone ICRF44, BD, Cat#555388, lot 9204345, dilution 1:50)
3. FITC Mouse Anti-Human CD64 (clone 10.1, Biolegend, Cat#305006, lot B256624, dilution 1:25)
4. APC Mouse Anti-Human CD80 (clone 2D10, Miltenyi Biotec, Cat#130-122-928, lots 5201106536, 5200113073, dilution 1:30)
5. BV421 Mouse Anti-Human CD163 (clone GHI/61, Biolegend, Cat#333612, lot B252819, dilution 1:50)
6. BV711 Mouse Anti-Human CD206 (clone 15-2, Biolegend, Cat#321136, lot B288972, dilution 1:50)
7. eFluor450 Mouse Anti-Human CD3 (clone OKT3, eBioscience, Cat#48-0037-42, lots 4342480, 2195904, 2169840, E08482-1634, dilution 1:200)
8. BV711 Mouse Anti-Human CD19 (clone HIB19, Biolegend, Cat#302245, 302246, lots B298219, B232488, B247856, dilution 1:50)
9. PE Mouse Anti-Human CD25 (clone 4E3, eBioscience, Cat#12-0257-41, lots 4278121, 2350756, dilution 1:50)
10. PerCP Mouse Anti-Human CD45 (clone 2D1, Biolegend, Cat#368505, 368506, lots B293458, B222147, B222149, dilution 1:50)
11. PE-CF594 Mouse Anti-Human CD56 (clone NCAM16.2, BD, Cat#564849, lots 9298555, 6028697, 1048114, dilution 1:100)
12. PE-Cy7 Mouse Anti-Human CD66b (clone G10F5, Biolegend, Cat#305116, 305115, lots B302355, B248575, B235705, dilution 1:200)
13. FITC Mouse Anti-Human CD69 (clone FN50, BD, Cat#555530, lot 9352977, dilution 1:50)
14. Alexa Fluor 700 Mouse Anti-Human CD8a (clone RPA-T8, Biolegend, Cat#301028, lots B210115, B330831, dilution 1:100)
15. APC-eFluor780 Mouse Anti-Human CD4 (clone OKT4, eBioscience, Cat#47-0048-42, lots 4309195, 2255511, dilution 1:50)
16. BV650 Mouse Anti-Human CD16 (clone 3G8, Biolegend, Cat#302042, lots B272554, dilution 1:200)
17. FITC Polyclonal Rabbit Anti-Human C1q Complement (Dako, Cat#F0254, lot 20061862, dilution 1:75)
18. BV785 Mouse Anti-Human CD45 (clone HI30, Biolegend, Cat#304048, dilution 1:100)
19. PE Mouse Anti-Human CD22 (clone S-HCL-1, BD, Cat#337899, dilution 1:20)
20. PE-CF594 Mouse Anti-Human CD19 (clone HIB19, BD, Cat#562294, dilution 1:200)
21. APC Mouse Anti-Human CD2 (clone RPA-2.10, eBioscience, Cat#17-0029-42, dilution 1:200)
22. FITC Rat Anti-Mouse C3 (clone RmC11H9, Cedarlane, Cat#C7503F, dilution 1:100)
23. Rat Anti-Mouse Mouse Fc block (clone 2.4G2, BD, Cat#553142, dilution 1:200)
24. Human Anti-Human Human Fc block (clone Fc1.3216, BD, Cat#564220, dilution 1:200)
25. FITC Mouse Anti-Human CD14 (clone MφP-9, BD, Cat#345784, dilution 1:50)
26. PE Mouse Anti-Human CD19 (clone J3-119, Beckman Coulter, Cat#A07769, dilution 1:50)
27. PE-Cy7 Mouse Anti-Human CD11c (clone Bu15, Biolegend, Cat#337215, dilution 1:400)
28. APC Mouse Anti-Human CD33 (clone WM53, BD, Cat#551378, dilution 1:400)
29. PerCP-eFluor710 Mouse Anti-Human CD56 (clone CMSSB, eBioscience, Cat#46-0567-41, dilution 1:50)
30. APC-eFluor780 Rat Anti-Mouse CD45 (clone 30-F11, eBioscience, Cat#47-0451-82, dilution 1:100)
31. Alexa Fluor 647 Rabbit HA Tag (Novus Biologicals, Cat#NB600-363AF647, 8 µg/ml)

Validation

For antibodies expressed recombinantly in-house, the concentration of purified IgGs was determined by absorbance at 280 nm. Quality assessment of purified antibodies was performed by SDS/PAGE (>90% intact IgG, >95% HC + LC under reducing conditions), ESI-TOF MS (identity confirmation), HP-SEC (aggregate level <5%) and binding assays on target-expressing cells (specificity). For in vivo studies, endotoxin levels were below 0.5EU/mg. For validation statements of commercially available antibodies we refer to the manufacturer's website.

Validation statements available for commercially accessible antibodies:
1. According to statements provided by the manufacturer, antibody M5E2-PE-Cy7 was reactive to human CD14 and validated for use in flow cytometry.
2. According to statements provided by the manufacturer, antibody ICRF44-PE was reactive to human CD11b and validated for use in flow cytometry.
3. According to statements provided by the manufacturer, antibody 10.1-FITC was reactive to human CD64 and validated for use in flow cytometry.
4. According to statements provided by the manufacturer, antibody 2D10-APC was reactive to human CD80 and validated for use in flow cytometry.
5. According to statements provided by the manufacturer, antibody GHI/61-BV421 was reactive to human CD163 and validated for use in flow cytometry.
6. According to statements provided by the manufacturer, antibody 15-2-BV711 was reactive to human CD206 and validated for use in flow cytometry.
7. According to statements provided by the manufacturer, antibody OKT3-eFluor450 was reactive to human CD3 and validated for use in flow cytometry by relative expression to ensure that the antibody binds to the antigen stated.
8. According to statements provided by the manufacturer, antibody HIB19-BV711 was reactive to human CD19 and validated for use in flow cytometry.
9. According to statements provided by the manufacturer, antibody 4E3-PE was reactive to human CD25 and validated for use in flow cytometry by cell treatment to ensure that the antibody binds to the antigen stated.
10. According to statements provided by the manufacturer, antibody 2D1-PerCP was reactive to human CD45 and validated for use in flow cytometry.
11. According to statements provided by the manufacturer, antibody NCAM16.2-PE-CF594 was reactive to human CD56 and validated for use in flow cytometry.
12. According to statements provided by the manufacturer, antibody G10F5-PE-Cy7 was reactive to human CD66b and validated for use in flow cytometry.
13. According to statements provided by the manufacturer, antibody FN50-FITC was reactive to human CD69 and validated for use in flow cytometry.
14. According to statements provided by the manufacturer, antibody RPA-T8-Alexa Fluor 700 was reactive to human CD8a and validated for use in flow cytometry.
15. According to statements provided by the manufacturer, antibody OKT4-APC-eFluor780 was reactive to human CD4 and validated for use in flow cytometry.
16. According to statements provided by the manufacturer, antibody 3G8-BV650 was reactive to human CD16 and validated for use in flow cytometry.
17. No validation statement available from the manufacturer.
18. According to statements provided by the manufacturer, antibody HI30-BV785 was reactive to human CD45 and validated for use

in flow cytometry.
19. According to statements provided by the manufacturer, antibody S-HCL-1-PE was reactive to human CD22 and validated for use in flow cytometry.
20. According to statements provided by the manufacturer, antibody HIB19-PE-CF594 was reactive to human CD19 and validated for use in flow cytometry.
21. According to statements provided by the manufacturer, antibody RPA-2.10-APC was reactive to human CD2 and validated for use in flow cytometry.
22. According to statements provided by the manufacturer, antibody RmC11H9-FITC was reactive to mouse C3 and validated for use in flow cytometry.
23. According to statements provided by the manufacturer, antibody 2.4G2 was reactive to mouse Fc and validated for use in flow cytometry.
24. According to statements provided by the manufacturer, antibody Fc1.3216 was reactive to human Fc and validated for use in flow cytometry.
25. According to statements provided by the manufacturer, antibody MϕP-9-FITC was reactive to human CD14 and validated for use in flow cytometry.
26. According to statements provided by the manufacturer, antibody J3-119-PE was reactive to human CD19 and validated for use in flow cytometry.
27. According to statements provided by the manufacturer, antibody Bu15-PE-Cy7 was reactive to human CD11c and validated for use in flow cytometry.
28. According to statements provided by the manufacturer, antibody WM53-APC was reactive to human CD33 and validated for use in flow cytometry.
29. According to statements provided by the manufacturer, antibody CMSSB-PerCP-eFluor710 was reactive to human CD56 and validated for use in flow cytometry.
30. According to statements provided by the manufacturer, antibody 30-F11-APC-eFluor780 was reactive to mouse CD45 and validated for use in flow cytometry.
31. According to statements provided by the manufacturer, antibody NB600-363AF647 was reactive to the HA tag and validated for use in immunofluorescence.

Eukaryotic cell lines

Policy information about cell lines

Cell line source(s)

Daudi, Raji, Ramos, COLO-205 and BxPC-3: American Type Culture Collection. U-698-M and REH: Deutsche Sammlung von Mikroorganismen und Zellkulturen. Wien-133 cells were kindly provided by Dr. Geoff Hale (BioAnaLab Limited, Oxford, UK).

Authentication

Cell line authentication was performed for Master Research Cell Banks of the cell lines Ramos, COLO-205, BxPC3 and Wien-133 using the CellCheck16 service provided by Idexx BioResearch. CellCheck16 consists of STR-based DNA profiling and multiplex PCR using 16 markers (15 STR loci and amelogenin) and comparison to profiles available in current public databases. The multiplex PCR assay detects the presence of DNA of human, mouse, rat, African green monkey or Chinese hamster. Identity testing reports are available upon request. B cell tumor cell lines Daudi, Raji, U-698-M and REH were characterized by flow-cytometry-based quantification of cell surface expression of human CD20, CD37, CD46, CD52, CD55, and CD59 relative to a calibration curve based on human IgG calibrator beads.

Mycoplasma contamination

All cell banks and cell cultures in use were routinely monitored for mycoplasma contamination during culturing, and tested negative for mycoplasma contamination.

Commonly misidentified lines
(See ICLAC register)

No commonly misidentified cell lines were used.

Animals and other organisms

Policy information about studies involving animals; ARRIVE guidelines recommended for reporting animal research

Laboratory animals

Specified in Methods section paragraph 'Pharmacokinetic analysis': 11-12 weeks old female tumor-free C.B17/IcrHan(r)Hsd-Prkdcscid mice; Methods section paragraph 'In vivo POC studies': female NSG mice (NOD.C.B-17-Prkdc scid/J mice; Charles River Laboratories) (8-11 weeks); female HPSC humanized NSG (NSG-HIS) mice (Jackson) (15-19 weeks). Mouse were housed under standard dark / light cycle (during the day 12 hrs light, night 12 hrs dark), in ambient temperatures (between 20 and 24 grade celsius) and humidity of between 45 and 65%.

Wild animals

The study did not involve wild animals.

Field-collected samples

The study did not involve field-collected samples.

Ethics oversight

As included in manuscript (paragraph 'Animals' in Methods section): Animal experiments were performed in compliance with the Dutch animal protection law (WoD) translated from the directives (2010/63/EU) and if applicable, the Code of Practice “animal experiments for cancer research” (Inspection V&W, Zutphen, The Netherlands, 1999) and were approved by the Dutch Central Committee for animal experiments and by the local Ethical committee. Animals were housed and handled in accordance with good animal practice as defined by the Federation of European Laboratory Animal Science Associations (FELASA), in an association for assessment and accreditation of laboratory animal care (AAALAC) and ISO 9001:2000 accredited animal facility (GDL, Utrecht, Netherlands).

Note that full information on the approval of the study protocol must also be provided in the manuscript.

Human research participants

Policy information about studies involving human research participants

Population characteristics	Population characteristics of blood samples or derivatives from healthy human donors were blinded to the authors in accordance with GDPR policy. Population characteristics of PBMCs derived from CLL patients are described in Supplementary Table 9. Importantly, this study did not involve any diagnostics specific to age, gender, or race, or draw any conclusions based on these patient characteristics.
Recruitment	PBMCs derived from CLL patients were commercially obtained from Discovery Life Sciences (Huntsville, AL, USA). Buffy coats from healthy human donors and complement-competent, pooled normal human serum (NHS; AB positive) were obtained from Sanquin (Amsterdam, The Netherlands). Whole blood samples from healthy human volunteers were freshly obtained from the University Medical Center Utrecht (Netherlands).
Ethics oversight	Commercially available patient-derived PBMCs and healthy donor blood(-derived) samples were collected at the site of the vendor after patient written and informed consent in accordance with the declaration of Helsinki. All vendors maintained strict ethical compliance, including fully de-identified materials and stringent Institutional Review Board (IRB) and Ethics Committee compliance.

Note that full information on the approval of the study protocol must also be provided in the manuscript.

Flow Cytometry

Plots

Confirm that:

☒ The axis labels state the marker and fluorochrome used (e.g. CD4-FITC).

☒ The axis scales are clearly visible. Include numbers along axes only for bottom left plot of group (a 'group' is an analysis of identical markers).

☒ All plots are contour plots with outliers or pseudocolor plots.

☒ A numerical value for number of cells or percentage (with statistics) is provided.

Methodology

Sample preparation	For an in-depth description of sample preparation, we refer to the individual assay sections within the online methods: cells and reagents, whole blood cytotoxicity, C1q binding, CDC, ADCP, FRET, and In Vivo POC studies.
Instrument	IntelliCyt iQue Screener flow cytometer (Sartorius) and LSRFortessa flow cytometer (BD Biosciences).
Software	FlowJo software v10.
Cell population abundance	N.A., as no cell sorting was performed.
Gating strategy	After gating of forward scatter (FSC) vs. side scatter (SSC), doublets were excluded by FSC-A vs. FSC-H. For whole blood assays, leukocytes were selected by CD45+ staining. Dead cells were excluded by fixable viability stain (FVS). Lymphocytes and granulocytes were separated based on CD66b staining. B- and T cells within the lymphocyte (CD66b-) cell population were identified as CD19+ and CD3+/(CD4+), respectively. More details are supplied in supplementary table 1 and supplementary figure 7.<br>For ADCP assays, target cells were distinguished from hMDM by CD11b, CalceinAM and CD19 staining as described in the 'ADCP' paragraph in the methods section. For binding assays, binding was quantified as the geometric mean fluorescent intensity (gMFI) of a directly-labeled antibody, or fluorochrome-conjugated secondary antibody respectively. More details are supplied in supplementary table 2 and supplementary figure 7.<br>For analysis of murine samples, gating and analysis are detailed in the relevant Methods Sections, documented in detail in supplementary table 8 and illustrated in supplementary figure 7.<br>For cytotoxicity assays, dead cells were distinguished by propidium iodide (PI) or fixable viability stain (FVS) as indicated.

☒ Tick this box to confirm that a figure exemplifying the gating strategy is provided in the Supplementary Information.
